# BK channels and alcohol tolerance: Insights from studies on *Drosophila*, nematodes, rodents and cell lines: A systematic review

**DOI:** 10.3892/mi.2025.232

**Published:** 2025-04-02

**Authors:** Luciana Angela Ignat, Raluca Oana Tipa, Alina Cehan Cehan, Vladimir Constantin Bacârea

**Affiliations:** 1 Doctoral School, ‘George Emil Palade’ University of Medicine, Pharmacy, Science and Technology of Targu Mures, 540142 Targu Mures, Romania; 2 ‘Prof. Dr. Alexandru Obregia’ Clinical Psychiatric Hospital, 041914 Bucharest, Romania; 3 Department of Psychiatry, ‘Carol Davila’ University of Medicine and Pharmacy, 020021 Bucharest, Romania; 4 Plastic and Reconstructive Surgery, Emergency Clinical County Hospital of Targu Mures, 540136 Targu Mures, Romania; 5 Department of Scientific Research Methodology, ‘George Emil Palade’ University of Medicine, Pharmacy, Science and Technology of Targu Mures, 540142 Targu Mures, Romania

**Keywords:** alcohol, tolerance, addiction, BK channels, *Drosophila*, nematodes, rodents, cell lines

## Abstract

Addictive disorders markedly affect the emotional, physical and financial wellbeing of individuals, placing a substantial burden on the healthcare system. With their widespread presence in the brain, large-conductance calcium and voltage-activated potassium (BK) channels play a crucial role in various aspects of neuronal function. They contribute to behavioral tolerance and are closely linked to neuronal activity and modulation through intracellular calcium levels. As such, BK channels serve as key models for investigating the mechanisms of the effects of alcohol. Investigating their role in alcohol tolerance provides a broader understanding of their physiological and pharmacological importance. The present systematic review examined the literature on the role of BK channels in alcohol tolerance and comprehensively explored the topic. For this purpose, two databases, Web of Science and PubMed, were searched, and studies published from 2000 until June, 2024 were included. After applying specific inclusion and exclusion criteria, 35 studies underwent analysis to present a chronological overview of BK channels and their relevance in alcohol tolerance development. The studies were categorized into four main groups, according to research conducted on: i) Fruit flies; ii) nematodes; iii) rodents; and iv) cell lines. Understanding the mechanisms through which alcohol interacts with these channels may help to elucidate the cellular and molecular mechanisms underlying alcohol tolerance. There is a growing interest in developing drugs that can precisely modulate BK channel activity to treat alcohol dependence and tolerance. However, additional studies are required to fully explain the complex mechanisms through which BK channels influence alcohol-related behaviors and to interpret these findings into clinical applications.

## Introduction

Large-conductance calcium and voltage-activated potassium (BK) channels are distinctive transmembrane proteins highly selective to potassium ions (K^+^). They regulate excitability, calcium signaling and potassium efflux across various species, from worms to mammals (1). Encoded by the single gene, potassium calcium-activated channel (KCN)MA1 (also known as Slo1), these channels help prevent hyperexcitability and potential cellular damage by promoting the extrusion of potassium, thereby maintaining cellular homeostasis. The regulation of K^+^ flow is essential for establishing and resetting the resting membrane potential, which in turn governs the action potential of excitable cells. The opening of BK channels results in a significant outflow of K^+^, leading to the hyperpolarization of the cellular membrane potential (2,3).

**Figure 1:**
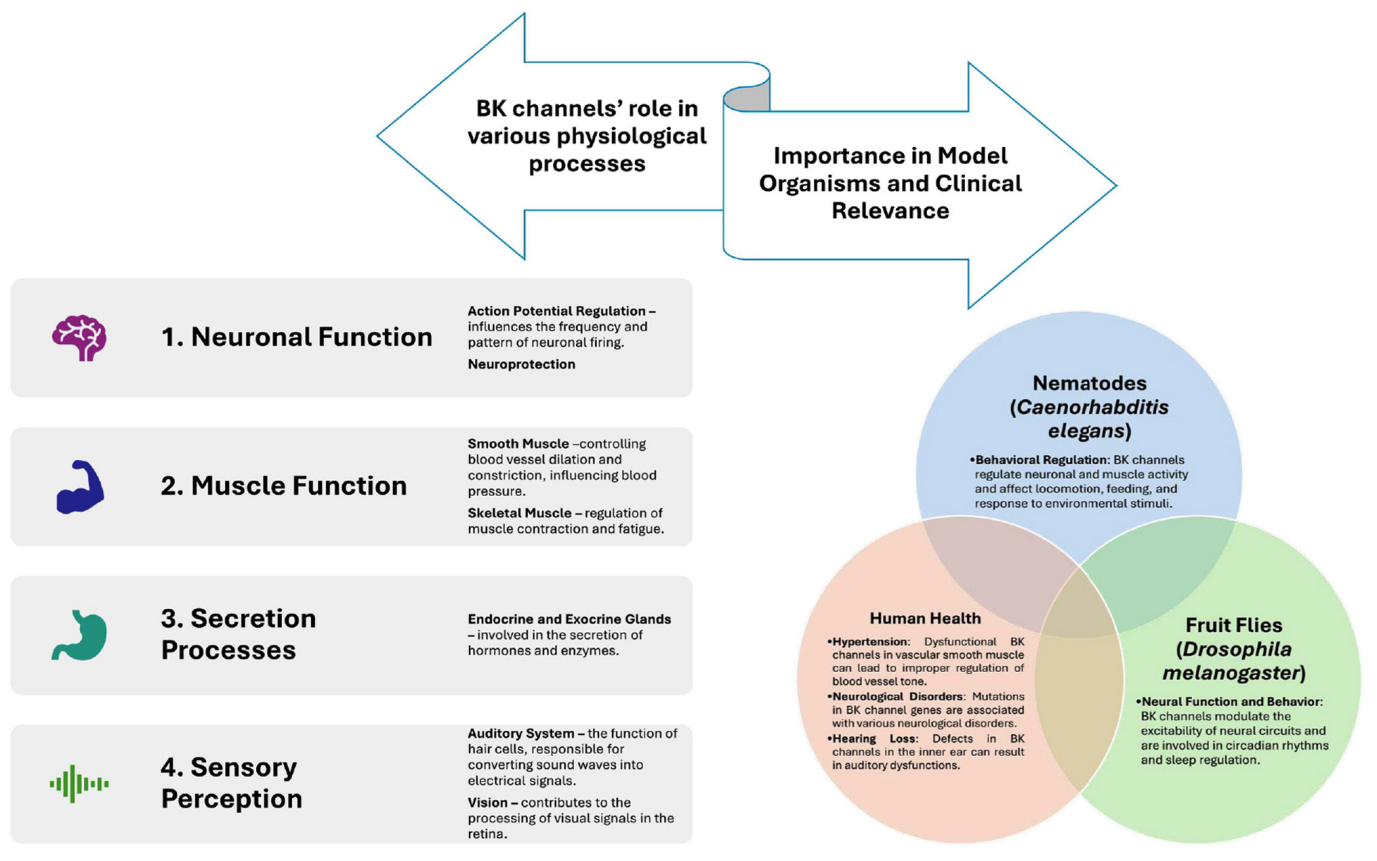
Role of BK channels in various physiological processes and their effects on nematodes, fruit flies and humans. BK channel, large-conductance calcium and voltage-activated potassium channel.

The K^+^ channel family comprises ≥79 genes in mammals that encode various subtypes of potassium channels, all characterized by a selectively permeable transmembrane pore for K^+^. BK channels, prevalent throughout the central nervous system (CNS), play vital roles in regulating circadian rhythms, blood flow, muscle contractions and renal excretion (4). When activated, these channels release K^+^, leading to hyperpolarization of the cellular membrane potential, which in turn modulates neuronal excitability and synaptic function. This process ultimately affects both motor skills and cognitive behavior (5). A summary of the role of BK channels in various physiological processes and their effects on nematodes, fruit flies and humans is presented in Fig. 1.

### BK channel subunits

The β subunits of BK channels are auxiliary proteins that regulate the activity of BK channels. These subunits, encoded by KCNMB, are categorized into four types: i) β1; ii) β2; iii) β3; and iv) β4. Each type exhibits unique tissue distribution and functional characteristics. The β1 subunit is expressed predominantly in smooth muscle cells, such as blood vessels, airways, and the urinary system, and is implicated in vasodilation and blood flow regulation. The β2 subunit, found mainly in the CNS, some endocrine cells and the ovaries, contributes to rapid signal processing in neurons, and may influence neuronal excitability and hormone secretion. The β3 subunit is involved in processes like spermatogenesis and insulin secretion. The β4 subunit is expressed at high levels in neurons of the CNS. Associated with neuronal adaptation and neuroprotection, it plays a critical role in alcohol tolerance and susceptibility to epilepsy. Each β subunit designs the function of the BK channel to meet the specific needs of the tissue in which it is expressed, making them critical for normal physiological processes and potential targets for therapeutic intervention (6,7).

The α subunit is the primary pore-forming component of BK channels. It consists of seven transmembrane segments (S0-S6), with the S0 segment being unique to BK channels. This subunit determines the basic properties of the channel, such as its conductance and sensitivity to voltage and calcium (8). BK channels can associate with auxiliary β subunits (β1-β4), which modulate the properties of the channel. The β4 subunit (KCNMB4) is primarily located in the brain and alters the gating properties of the channel, regulating neuronal excitability and protecting against excessive activity. The effects of the β subunits on ethanol (EtOH)-induced potentiation of BK channels vary. In mice, under chronic intermittent EtOH conditions, β1 deficiency increases EtOH consumption during withdrawal, while β4 absence reduces it (5,8).

### BK channels and alcohol use disorder (AUD)

EtOH facilitates the activation of BK channels at neuro-hypophysial terminals, thereby modulating the release of vasopressin and oxytocin. However, it does not affect BK channels in the cell body and dendrites (9). It may also influence BK channel movement to the cell surface, leading to increased internalization within 3-6 h and contributing to alcohol tolerance. It is unclear whether this interaction directly causes intoxication or chronic tolerance; however, behavioral evidence supports both possibilities (10,11). Both acute and chronic alcohol exposure affect synaptic plasticity and influence synaptic transmission efficacy (11).

EtOH affects BK channels and G-protein-gated rectifying potassium channels in the brain, which regulate responses in the reward circuit and may be potential targets for treating AUD (12,13). Additionally, EtOH increases the expression of a two-pore potassium channel (KCNK12) in the ventral tegmental area (VTA) and its knockdown increases alcohol consumption (14). Mulholland *et al* (15) investigated how EtOH exposure induces plasticity in neural circuits, which is crucial for understanding the way alcohol affects the brain over short- and long-term periods. This plasticity underlies the development of tolerance and addiction.

AUD is a multifaceted neurological condition believed to stem from a combination of genetic, epigenetic and environmental influences. These factors contribute to the development of patterns of excessive alcohol consumption and dependence (16-18). Research has indicated that changes in KCa^2+^ channels are responsible for the flexibility of the ability of the brain to generate electrical signals and may influence the drive to seek alcohol, particularly during periods of abstinence (19,20).

Recent research has revealed explicit connections between the modulation of BK channels and AUD. BK channels are expressed in key brain regions implicated in reward processing and addiction, such as the nucleus accumbens and the VTA. The interaction of alcohol with BK channels in these areas can enhance dopamine release, reinforcing alcohol-seeking behaviors and contribute to neuroadaptive changes that underlie addiction (19,20).

Chronic alcohol consumption leads to changes in BK channel subunit expression and sensitivity, contributing to alcohol dependence. Polymorphisms in genes encoding BK channel subunits have been associated with susceptibility to AUD in specific populations. Variants that modify BK channel activity may influence individual responses to alcohol and predispose individuals to problematic use (5,21).

During alcohol withdrawal, the reduced BK channel function contributes to neuronal hyperexcitability and seizure susceptibility. This suggests a role for BK channels in managing withdrawal symptoms and the risk of relapse. BK channels are involved in stress responses and are often co-factors in the development and persistence of AUD. The dysregulation of these channels may exacerbate stress-induced drinking behavior (22).

BK channel activators are being explored to counteract hyperexcitability during withdrawal or reduce alcohol-induced euphoria. In some contexts, BK channel blockers may reduce the rewarding effects of alcohol, potentially curbing addiction. Studies using rodents have demonstrated that the pharmacological manipulation of BK channels alters alcohol consumption patterns and withdrawal severity (22,23). Electrophysiological recordings demonstrate that the effects of alcohol on neuronal excitability are mediated partly through BK channel modulation (24).

The fruit fly, *Drosophila melanogaster*, is a potent model organism due to its genetic tractability, short life cycle and availability of sophisticated genetic tools. Advanced genetic techniques in *Drosophila* allow for the precise manipulation of genes and pathways, providing clear causal links between genetic changes and phenotypic outcomes related to drug tolerance and dependence. The study by Ghezzi and Atkinson (21) explored the role of ion channels and receptors in mediating homeostatic responses to drugs. Alterations in ion channel function and receptor signaling pathways are identified as key components of adaptive responses to drug exposure (24-26).

In model organisms such as fruit flies, manipulating BK channel activity through genetic modification or pharmacological agents affects EtOH-related behaviors, such as locomotion, sedation and tolerance. Similar findings have been observed in rodents, where BK channel function influences alcohol consumption patterns, motor coordination and susceptibility to the effects of EtOH (27,28). In humans, genetic variations in the BK channel genes have been found to be associated with differences in alcohol sensitivity and susceptibility to AUD. Evidence from human studies suggests that drugs affecting BK channels can alter alcohol-related behaviors and potentially serve as therapeutic agents for the management of AUD (29-31).

Over the past 10 years, there has been a surge in knowledge about BK channels, including their expression, alcohol sensitivity and potential impact on neuronal activity in various brain regions such as the striatum (32). The genetic screening of *Caenorhabditis elegans* (*C. elegans*) has revealed that mutations in the Slo-1 gene led to resistance to EtOH intoxication. This gene encodes a subunit of a K^+^ channel that limits excitatory neurotransmitter release. EtOH potentiates Slo-1, causing intoxication by reducing neurotransmitter release (33,34).

AUD is associated with marked medical, neurological and psychiatric comorbidities, leading to increased morbidity and mortality (35). At present, there are only some FDA-approved treatments for AUD, and their effectiveness in lowering relapse rates tends to be relatively limited (36-38). Research on gene expression changes should be considered preliminary and further validation experiments are thus required. However, data from drug interventions and knockout animal models can provide insight into potential causal associations. Recent studies using rodent models of AUD have identified variations in genes encoding K^+^ channels in the brains of animals with different alcohol intake levels. Notably, FDA-approved potassium channel regulators have been found to decrease alcohol intake in rats. Understanding the mechanisms through which alcohol interacts with these channels may help to elucidate the cellular and molecular mechanisms underlying alcohol tolerance (39-41).

AUD involves both psychological factors, such as cravings, anxiety and stress, and biological factors such as changes in brain chemistry and neural circuitry (41). BK channels represent a point of convergence where these perspectives can be integrated. By studying BK channel activity, the way biological changes contribute to psychological experiences associated with alcohol use and addiction can be understood, providing a holistic view of the way these disorders develop and persist. BK channels are being explored as a potential therapeutic target for AUD. Understanding the contribution of these channels to alcohol tolerance is necessary for devising strategies to modulate BK channel activity, with the potential to mitigate tolerance and dependence. This translational approach, spanning from fundamental biological research to the development of potential treatments, represents an initial step in addressing this issue from a psychobiological standpoint.

In the present systematic review, the aim was to analyze the studies published in the literature regarding the implications of BK channels in developing alcohol tolerance. A detailed and complex understanding of BK channels and their role in alcohol tolerance is essential for advancing scientific knowledge, developing effective treatments and improving public health outcomes related to alcohol use and dependence.

## Materials and methods

### Search strategy

A literature search was conducted using two online databases: i) MEDLINE (accessible through PubMed); and ii) Web of Science Core Collection. This approach enabled the gathering of all relevant articles published from 2000 up until June, 2024. The key words used for the search were as follows: ‘bk channels’ OR ‘large conductance calcium-activated potassium channels’ AND ‘alcohol tolerance’.

### Study selection and data extraction

**Figure 2:**
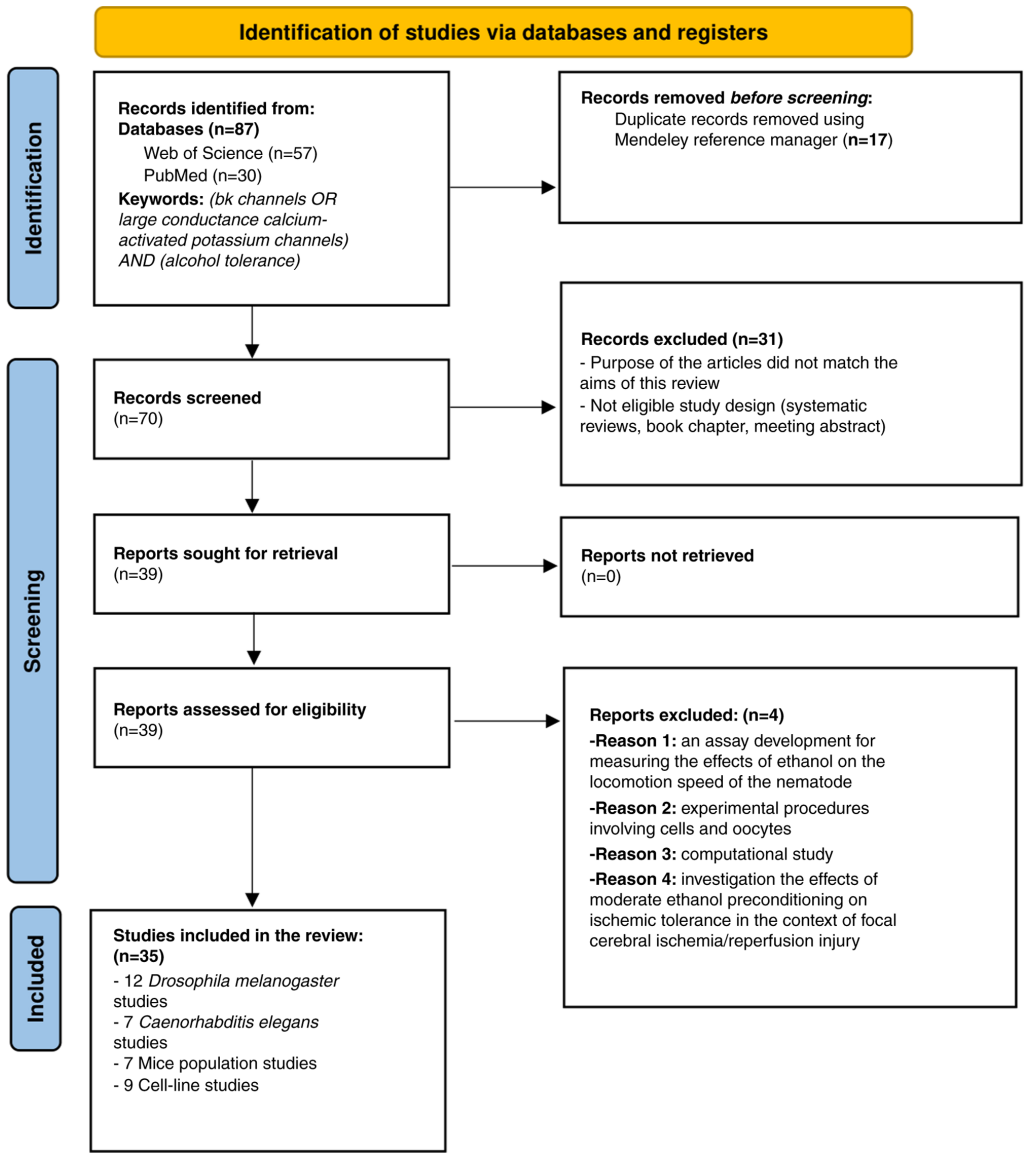
PRISMA flow diagram for search outcomes and screening process.

During the manual selection process, examined titles, abstracts and full-text articles were carefully examined to assess their relevance to the topic. Of note, two authors independently categorized the articles identified according to established inclusion and exclusion criteria. Any discrepancies in their evaluations were resolved through discussions with a third author. This selection process adhered to the guidelines set forth by PRISMA (Fig. 2) (42). Additionally, any duplicate entries were automatically eliminated using the Mendeley Reference Manager version 2.130.2.

### Inclusion criteria

Studies were deemed suitable for inclusion in the systematic review if they met all of the following criteria: i) The title contained the relevant key words (bk channels, large conductance calcium-activated potassium channels, alcohol tolerance); ii) titles were at least somewhat related to the key words; iii) articles were either written in English or in other languages, provided that abstracts and full texts were translated into English; iv) abstracts were accessible; v) full texts were available; vi) there was a minimum level of outcomes and statistical analysis (including descriptive and/or inferential statistics, significance tests such as t-tests, ANOVA, or χ^2^ tests, regression, correlation analysis, or probability distributions); vii) the studies were experimental; and viii) the articles represented original research focused on the implications of BK channels in alcohol tolerance.

### Exclusion criteria

Articles were excluded from the present study based on several criteria: i) Inappropriate study design or article format; ii) lack of relevance to the implications of BK channels in alcohol tolerance; iii) absence of an available abstract; iv) unavailability of the full text in English; and v) inclusion of computational studies. After determining eligibility, the remaining articles underwent a thorough full-text analysis. This thorough evaluation covered various aspects, including the objectives of each study, the animal models used, the interventions applied and the main findings.

### Quality assessment and risk of bias

**Figure 3:**
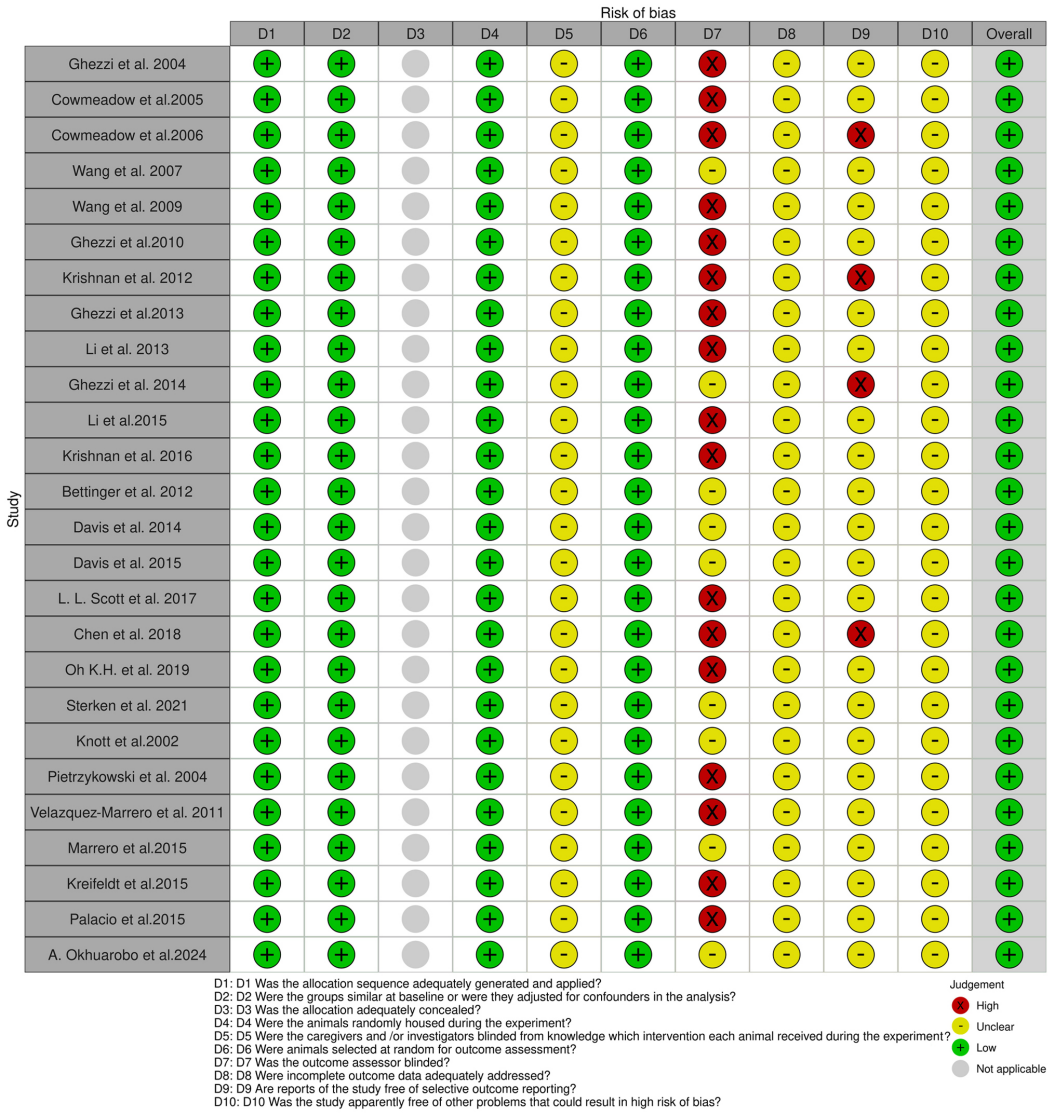
SYRCLE tool for assessing the risk of bias. The studies listed are as follows: Ghezzi *et al* (57), Cowmeadow *et al* (59), Cowmeadow *et al* (60), Wang *et al* (62), Wang *et al* (64), Ghezzi *et al* (66), Krishnan *et al* (68), Ghezzi *et al* (69), Li *et al* (71), Ghezzi *et al* (73), Li *et al* (74), Krishnan *et al* (76), Bettinger *et al* (81), Davis *et al* (82), Davis *et al* (84), Scott *et al* (48), Chen *et al* (86), Oh *et al* (87), Sterken *et al* (89), Knott *et al* (93), Pietrzykowski *et al* (95), Velázquez-Marrero *et al* (97), Marrero *et al* (98), Kreifeldt *et al* (102), Palacio *et al* (100), Okhuarobo *et al* (101).

**Figure 4:**
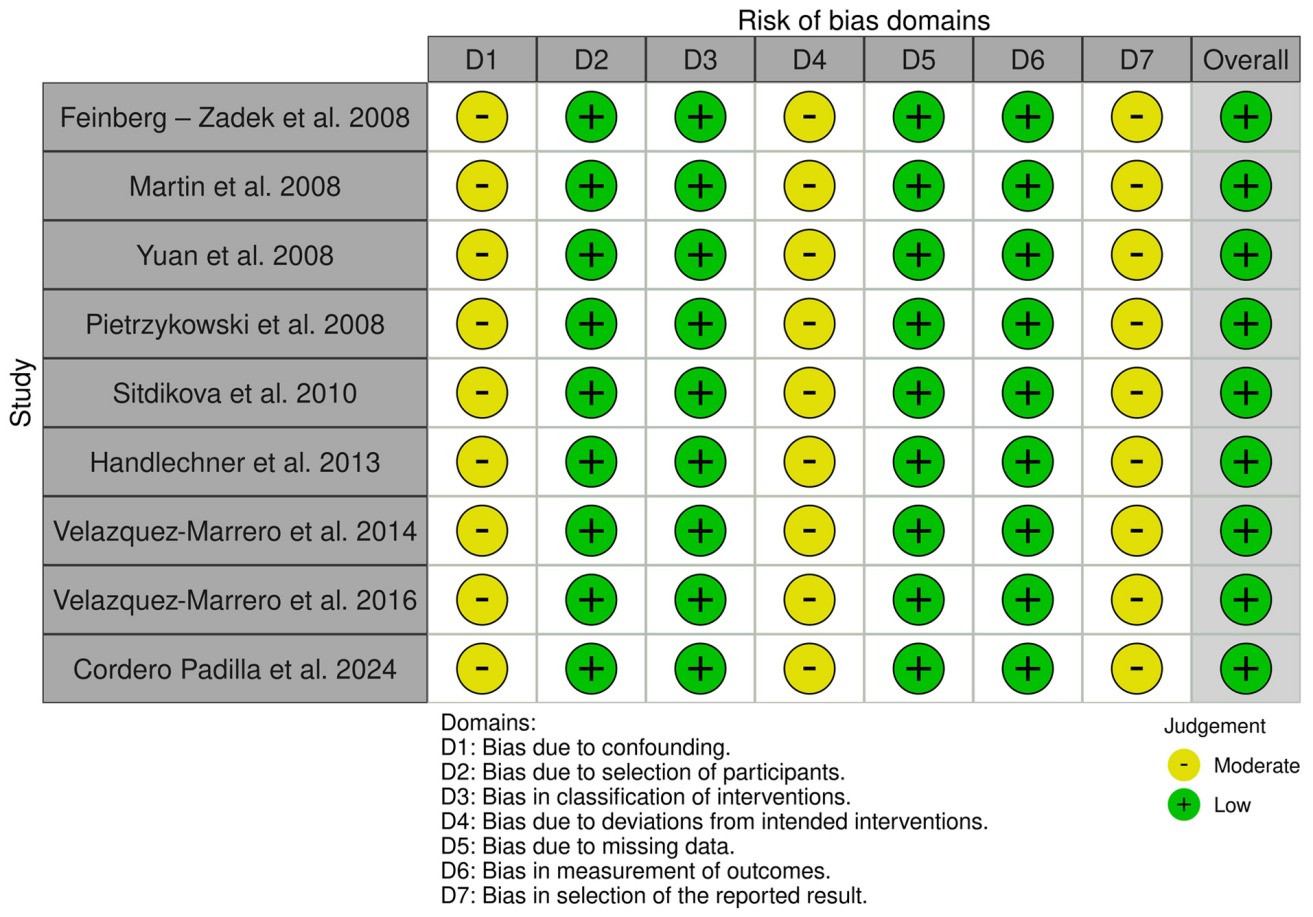
Risk of bias in non-randomized studies-of interventions (ROBINS-I). The studies listed are as follows: Feinberg-Zadek *et al* (103), Martin *et al* (104), Yuan *et al* (106), Pietrzykowski *et al* (107), Sitdikova *et al* (108), Handlechner *et al* (109), Velázquez-Marrero *et al* (110), Velázquez-Marrero *et al* (111), Cordero Padilla *et al* (112).

The Systematic Review Centre for Laboratory Animal Experimentation (SYRCLE) online tool version 0.3.0.900 was used to evaluate potential biases in 26 animal model studies. The resulting visualizations, which illustrate the risk of bias, are presented in Fig. 3. Additionally, the risk of bias in cell-line studies was assessed using the ROBINS-I online tool 2016 version, to evaluate risk in nonrandomized studies of interventions (Fig. 4) (43). The tool is available at https://mcguinlu.shinyapps.io/robvis/.

A ‘low’ risk of bias indicates high confidence in the results of a study due to strong methodological safeguards. By contrast, a ‘high’ risk of bias points towards significant reliability concerns stemming from weaknesses in design or execution. Blinding in animal studies is often poorly implemented, leading to detection or performance bias and resulting in ‘unclear risk of bias’ according to the SYRCLE tool. Despite the ‘high’ risk in certain areas, the findings of a study can still be valuable. Overall risk is assessed by combining domain ratings and considering the study context, with mainly ‘low risk’ ratings indicating reliability (44-46). The overall risk of bias for studies in the present systematic review was ‘low’, with a detailed analysis presented in Table I.

## Results

The initial search identified 87 potentially relevant articles from PubMed and Web of Science. The Mendeley reference manager efficiently eliminated 17 duplicates. During the screening phase, 70 articles were analyzed based on their titles and abstracts, excluding 31 articles due to missing abstracts, ineligible study designs, or a mismatch with the overall aim of the review. The full texts were then reviewed to clarify the relevance of those abstracts that lacked sufficient information. Following these steps, the eligibility of 39 articles was assessed. However, four clinical studies were excluded as they did not specifically examine the role of BK channels in the mechanism of alcohol tolerance. Ultimately, 35 articles were identified which met the inclusion criteria, including studies on 12 *Drosophila melanogaster* populations, seven *C. elegans*, seven rodent populations and nine cell lines. A detailed analysis of their full texts was conducted. A summary of the included studies is presented in Tables II, III, IV and V, providing an easy-to-read reference for readers to interpret the findings chronologically. The present systematic review protocol has been registered in OSF Registries, and the published version can be accessed at https://osf.io/hv2jz (last accessed on October 30, 2024).

## Discussion

*Mechanisms of tolerance shared across organisms.* The BK channels have become an essential molecular mechanism underlying addiction-related behaviors across multiple species, including *Drosophila melanogaster* (fruit flies), *C. elegans* (nematodes) and rodents. While there are species-specific variations in the mechanisms and behavioral outcomes, studies suggest evolutionary conservation in the role of BK channels in modulating neural excitability, reward pathways and responses to addictive substances (22,26,29-33).

EtOH tolerance involves similar signaling pathways across species, such as changes in neurotransmitter systems (GABAergic and glutamatergic systems) and ion channel modulation. Cellular adaptations, such as altered gene expression and protein phosphorylation, are conserved mechanisms that mediate alcohol tolerance. Mutations in the Slo gene lead to impaired tolerance in *Drosophila melanogaster*, as BK channel activity regulates neural excitability during repeated EtOH exposure. EtOH exposure increases Slo expression in specific neurons, suggesting a role in adaptation to substance exposure. Slo mutants show altered locomotion and EtOH-induced sedation behaviors, linking the BK channels to motor control and addiction-like behaviors (22,39).

Slo-1 channels reduce the synaptic release of neurotransmitters, stabilizing neural activity and preventing over-excitation associated with drug exposure in *C. elegans*. Mutant nematodes lacking functional Slo-1 exhibit abnormal movement and altered responses to environmental cues under the influence of drugs. BK channels encoded by KCNMA1 are similarly implicated in EtOH tolerance and withdrawal in mammals. Mice with an altered KCNMA1 function exhibit changes in alcohol preference, consumption and withdrawal severity (47).

Across all models, chronic EtOH exposure induces gene expression changes to adapt cellular functions. Transcription factors, such as myocyte enhancer factor 2 and cAMP response element-binding protein (CREB) regulate synaptic plasticity genes in flies and rodents. In cell lines, EtOH-induced epigenetic modifications affect stress response and metabolism-related genes. In nematodes, gene transcriptional changes like Slo-1 contribute to tolerance (48).

Locomotor changes, such as impaired movement or sedation following acute EtOH exposure, are observed in flies, nematodes and rodents, reflecting conserved neural responses to alcohol. Repeated EtOH exposure leads to tolerance (reduced behavioral effect) in all groups. Across all three species, BK channels regulate the balance of excitation and inhibition in the nervous system, a critical factor in drug-induced plasticity. Their role in adapting neural circuits to repeated substance exposure highlights a conserved mechanism in managing neural stress and reward. While flies and worms provide insight into the genetic and cellular mechanisms, mice offer a model closer to humans for studying complex addiction-related behaviors (22).

### Central role of neuronal plasticity

Alcohol exposure induces neural plasticity in all these organisms. For example, in *C. elegans*, alcohol affects neurotransmission by modulating GABAergic signaling and synaptic plasticity. In mice, chronic alcohol exposure alters synaptic strength, contributing to tolerance. Also, EtOH influences protein kinases (PKs), such as PKA and PKC across species, affecting ion channels and synaptic proteins. These signaling pathways are critical in *C. elegans*, *Drosophila* and mammalian cells for the development of tolerance (49,50).

*Behavioral responses.* In *Drosophila*, EtOH exposure leads to hyperactivity, followed by sedation and tolerance develops with repeated exposure. In *C. elegans*, EtOH attenuates movement and impairs egg-laying, with tolerance evident as recovery in these behaviors. Behavioral responses in mice include sedation, ataxia and eventually tolerance, with changes observed at molecular and circuit levels. In cell lines, studies focus on isolated molecular pathways, such as BK channel function or GABA receptor modulation without the influence of whole-organism dynamics (31,34,35,51).

*Cellular stress responses.* EtOH induces cellular stress across all systems. The upregulation of heat-shock proteins (HSPs) is observed in flies, nematodes, mice and cell lines, helping stabilize proteins and mitigate damage. The unfolded protein response pathway, primarily studied in cell lines and mice, is a conserved mechanism to protect cells from EtOH-induced ER stress (52).

*Metabolic adaptations.* Enzymatic pathways for EtOH metabolism involving alcohol dehydrogenase (ADH) and aldehyde dehydrogenase are conserved in flies, mice and some cell lines. While nematodes have limited alcohol metabolism, they still exhibit compensatory adaptations at the cellular level. Enhanced alcohol metabolism reduces EtOH availability and toxicity, contributing to tolerance (53).

*Membrane adaptations.* EtOH disrupts lipid bilayers in all systems. Chronic exposure alters membrane composition to stabilize neuronal and cellular function. In flies and cell lines, adaptations in lipid bilayers reduce the effects of EtOH on membrane proteins. Similar mechanisms are inferred in rodents and nematodes, although these are more pronounced in complex organisms (54,55).

*Differences among organisms. C. elegans* has a simple nervous system with 302 neurons, which renders the study of the genetic and molecular mechanisms easier, although it limits behavioral complexity. *Drosophila* has a more complex brain, allowing more nuanced behaviors, such as associative learning in response to alcohol. Mice have a highly complex nervous system and display a wide range of alcohol-related behaviors, including addiction-like symptoms and social interactions. Cell lines lack neural networks, so they serve as models for studying isolated cellular and molecular mechanisms, like BK channel function, in response to EtOH (24-26).

*C. elegans* and *Drosophila* are genetically tractable, allowing high-throughput genetic screens. Mice are less amenable to large-scale genetic manipulation but provide a closer model of human physiology. Cell lines provide unlimited manipulations of individual genes or pathways without whole-organism complexity (27,28,54).

Environmental factors, such as temperature and humidity markedly influence alcohol responses in *Drosophila* and *C. elegans*. In mammals, metabolic factors such as liver enzymes, for example ADH, play a critical role in alcohol processing. The effective concentration of EtOH required to modulate BK channels and elicit behavioral changes is typically lower in fruit flies due to their smaller size and metabolic differences compared to rodents. In simpler models (*C. elegans* and *Drosophila*), BK channel modulation primarily affects basic behaviors such as locomotion. In mice, BK channels play a role in more complex behaviors, including withdrawal and addiction-like processes (29,55).

### Chronological presentation of Drosophila melanogaster, C. elegans, rodents and cell line studies

Numerous studies have provided notable insight into the effects of alcohol on the nervous system by investigating the interaction between BK channels and alcohol in various organisms and cell lines. Studies on genetics and electrophysiology have consistently indicated the marked influence of these channels on alcohol sensitivity, tolerance and adaptive plasticity. As such, they have become a focal point in alcohol research (56).

*Drosophila melanogaster studies. Drosophila melanogaster*, commonly known as the fruit fly, is used as a model organism in studying alcohol use and its effects due to its genetic similarity to humans. The fruit fly exhibits behavioral responses to alcohol analogous to those observed in mammals, including humans and has a short life cycle, allowing for rapid data generation and longitudinal studies over multiple generations in a relatively short period (12). The present systematic review presents 12 studies conducted on fruit flies from 2004 to 2016; these are as follows:

In 2004, Ghezzi *et al* (57) conducted a study using mutants and transgenic flies to manipulate the expression of the Slo gene, which codes for the BK channel. To induce rapid tolerance, the flies were exposed to benzyl alcohol. Upon exposure to benzyl alcohol, *Drosophila* exhibited a rapid and significant increase in Slo gene expression within their nervous system. Conversely, mutant flies with a disrupted Slo gene did not develop tolerance. Electrophysiological recordings confirmed that neurons from flies with heightened Slo expression displayed an elevated potassium current, likely contributing to their reduced sensitivity to sedative drugs. These findings provide insight into the molecular mechanisms underlying drug tolerance and identify potential targets for addressing issues related to drug abuse and addiction (58). In a following study, Cowmeadow *et al* (59) used wild-type *Drosophila* and a range of Slo mutants, such as null mutants without functional Slo genes and hypomorphic mutants with diminished Slo function, to trigger acute and chronic EtOH tolerance. Transgenic flies with inducible Slo expression exhibited a heightened tolerance when this gene expression was elevated, indicating that heightened BK channel activity plays a role in EtOH tolerance.

In 2006, it was shown that wild-type *Drosophila* developed notable EtOH tolerance after repeated exposures, as indicated by reduced sedation times upon subsequent EtOH exposures (60). Conversely, mutant strains with impaired SLO genes did not exhibit the same tolerance level. Furthermore, flies with inducible SLO expression demonstrated faster recovery times and increased resistance to EtOH-induced sedation than control flies. Understanding the molecular pathways involved in EtOH tolerance could be instrumental in the development of targeted therapies for managing alcohol dependence and withdrawal. This knowledge has the potential to enhance treatment outcomes for individuals with AUD (60,61).

In another study, Wang *et al* (62) sought to elucidate the underlying molecular mechanisms responsible for enduring changes in gene expression and behavior following repeated drug exposure. Their focus centered on the influence of histone modifications and DNA methylation, and employed the loss of righting reflex assay to gauge the development of EtOH tolerance. Analyses of chromatin immunoprecipitation assays indicated that exposure to EtOH resulted in elevated levels of histone acetylation at the promoters of genes associated with drug tolerance. These modifications are typically indicative of active transcription. Furthermore, examination of DNA methylation patterns revealed alterations in response to EtOH exposure, particularly in genes related to the regulation of synaptic function and neural plasticity. *Drosophila melanogaster* with mutations in genes involved in histone modification and DNA methylation exhibited compromised development of EtOH tolerance (63).

In 2009, the study by Wang *et al* (64) aimed to understand the molecular mechanisms by which CREB modulates BK channel gene expression in response to drug exposure. Mutant fly strains with modified CREB binding sites in the SLO promoter were utilized to evaluate the functional significance of CREB regulation. Chromatin immunoprecipitation assays revealed the binding of CREB to the promoter region of the SLO gene, indicating direct regulatory involvement. Exposure to EtOH resulted in elevated expression of the SLO gene, and this upregulation was determined to be facilitated by CREB, as evidenced by reduced SLO expression in flies with impaired CREB activity. The binding of CREB to the SLO promoter enhances gene expression, thereby contributing to physiological adaptations associated with the development of tolerance (65).

In the study by Ghezzi *et al* (66), the primary objective was to investigate the role of BK channels in the development of drug tolerance and dependence, focusing on their influence as counter-adaptive mechanisms. Flies with reduced BK channel activity exhibited an increased sensitivity to EtOH-induced sedation. Conversely, flies with overexpressed BK channels were more resistant to sedation. Withdrawal symptoms were more severe in flies with reduced BK channel activity, indicating a heightened state of dependence. In addition, BK channels were identified as applying a counter-adaptive function, antagonizing the development of tolerance. The overexpression of these channels was associated with a reduction in tolerance development. Furthermore, the induced elevation of slowpoke gene expression resulted in replicating the tolerant phenotype. This implies that modulating BK channel activity has the potential to mimic the physiological adjustments observed in drug tolerance (67).

Krishnan *et al* (68) concluded in 2012 that dynamin is essential for developing EtOH tolerance in *Drosophila*. By regulating endocytosis and vesicle trafficking, dynamin influences the neuronal adaptations required for tolerance. Subsequently, 1 year later, Ghezzi *et al* (69) identified several neuronal populations as critical for developing EtOH tolerance. Key regions included the mushroom bodies and specific neurons within the central brain. Their study provided a detailed map of the neurons and genetic pathways of *Drosophila* involved in EtOH tolerance. The research advanced the understanding of the neural and molecular mechanisms underlying drug tolerance by demonstrating the roles of specific neurons and the cAMP signaling pathway (70).

Li *et al* (71) identified a specific DNA element within the *Drosophila* genome that plays a crucial role in regulating the expression of genes associated with drug tolerance and withdrawal. The functional analysis results indicated that the identified DNA element plays a vital role in regulating the expression of the SLO gene. Altering this DNA element led to notable changes in the behavioral responses of the flies to EtOH (72). Specifically, modifications to the DNA element affected the development of drug tolerance and the manifestation of withdrawal symptoms. This study employed transgenic flies to illustrate that changes in the regulatory DNA element had a direct impact on the behavioral traits associated with drug tolerance and withdrawal (71).

The 2014 study by Ghezzi *et al* (73) investigated the role of BK channel gene expression in the susceptibility of *Drosophila* to EtOH withdrawal seizures. It was found that flies with elevated levels of BK channel expression experienced more severe withdrawal seizures, which was confirmed through behavioral assays. In 2015, the researchers identified a specific histone modification (H3K4me3) associated with active gene transcription at the SLO BK channel gene locus in *Drosophila* muscle tissue. It was suggested that specific transcription factors bind to this regulatory DNA element, facilitating the recruitment of the transcriptional machinery and histone modifications that activate gene expression (74). A particular element of DNA in the 5' flanking region of the SLO gene was found to be crucial for its expression in muscle tissue. These findings have broader implications for epigenetics, highlighting the way specific histone modifications and DNA elements control gene expression (74,75).

Subsequently, a specific DNA element within the SLO gene was identified that modulates EtOH tolerance in *Drosophila*. Behavioral assays were administered to evaluate EtOH tolerance in flies with and without the intact DNA element. Flies with a disrupted or deleted regulatory element exhibited a significantly diminished ability to develop tolerance to EtOH compared to control flies. Flies lacking this regulatory element displayed altered SLO gene expression, corresponding to impaired EtOH tolerance (76,77).

*C. elegans studies.* *C. elegans* is a tiny, transparent nematode (roundworm) that has become a potent model organism in biological research. Its simplicity, well-characterized biology and extensive genetic tools render it ideal for studying various biological processes, including the function of ion channels, such as BK channels and the effects of alcohol (78). Nematodes possess a relatively simple nervous system comprising 302 neurons, rendering them an advantageous model for investigating neuronal circuits and behaviors. Alcohol exposure induces changes in *C. elegans* behaviors, including alterations in locomotion, egg-laying and feeding patterns (79). Moreover, genetic studies involving *C. elegans* mutants with modified BK channel function have demonstrated varying sensitivities to alcohol. These findings contribute to the elucidation of the genetic and molecular mechanisms that underlie alcohol sensitivity and tolerance, thereby offering potential targets for therapeutic interventions related to AUD (79).

The short lifecycle of *C. elegans* (~3 days from egg to adult) enables quick data generation and long-term studies across multiple generations. It is also easily manipulated genetically, allowing for precise control and observation of gene function. These are only some of the advantages of using *C. elegans* in BK channel and alcohol research (80). A number of genetic pathways in *C. elegans* are conserved across higher organisms, including humans. Thus, discoveries in *C. elegans* frequently relate to understanding human biology and diseases. The following eight studies conducted on nematodes will be analyzed.

In 2012, it was found that altering the lipid composition of *C. elegans* affected the development of acute tolerance to EtOH (81). Mutants with alterations in genes involved in lipid metabolism displayed different levels of acute EtOH tolerance compared to wild-type *C. elegans*. Behavioral assays demonstrated that changes in the lipid environment affected the locomotion of *C. elegans* in response to EtOH. Wild-type and mutant worms exhibited varying degrees of reduced movement after EtOH exposure, associated with their lipid composition (81). Supplementation of the diet with polyunsaturated fatty acids enhanced the development of acute EtOH tolerance. Conversely, supplementation with saturated fatty acids impaired the development of tolerance. Demonstrating that lipid composition can influence tolerance development, that study provides insight into the tolerance molecular mechanisms of EtOH, which could have broader implications for understanding similar processes in higher organisms, including humans (81).

Subsequently, 2 years later, Davis *et al* (82) aimed to pinpoint a specific amino acid residue in the BK potassium channel that is necessary for the activation of the channel by alcohol and to determine the mechanisms through which this residue contributes to alcohol-induced behavioral changes, specifically intoxication. It was found that a specific missense mutation, T381I, in the RCK1 domain of SLO-1 made the nematodes highly resistant to alcohol intoxication. This mutation did not affect other behaviors dependent on the BK channel, indicating that the mutant channel retained its normal function *in vivo*. Patch-clamp recordings revealed that the human BK channel carrying the T352I mutation exhibited insensitivity to alcohol-induced activation (82). However, the mutation did not affect the average conductance and potassium selectivity of the channel, and only minor deviations in voltage dependence were observed (83).

In 2015, mutations in *C. elegans* were examined, focusing on disrupting the calcium-binding sites of the BK channel (SLO-1). A previous study yielded findings indicating that the presumed calcium-binding domains of the BK channel in *C. elegans* do not play a crucial role in the activation of the channel by EtOH or EtOH-induced intoxication (84). According to Scott *et al* (48), *C. elegans* exhibited notable behavioral deficits following withdrawal from chronic EtOH exposure, including reduced locomotion speed and altered movement patterns. Mutant strains lacking functional SLO-1 channels showed exacerbated behavioral deficits following EtOH withdrawal compared to wild-type strains. Overexpression of SLO-1 channels mitigated some of the behavioral deficits associated with EtOH withdrawal. The proper functioning of SLO channels is essential for average behavioral recovery, providing valuable insight into the molecular mechanisms underlying EtOH-related withdrawal effects (49,85).

In 2018, Chen *et al* (86) identified the GCY-35/GCY-36 and TAX-2/TAX-4 signaling pathways as mediators of acute functional tolerance to EtOH. Mutants with disruptions in these pathways exhibited impaired development of acute EtOH tolerance and reduced behavioral adaptation to EtOH, providing insight into the molecular and neuronal mechanisms of the exposure to EtOH. It is essential to mention that these pathways are involved in the oxygen sensory neurons, which affect the mechanisms through which nematodes respond to EtOH. According to the studies by Oh and Kim (87) and Oh *et al* (88), BK channels need to be organized into clusters to function correctly in the presence of EtOH, and mutations which disrupt the clustering of BK channels result in altered behavioral sensitivity to EtOH. These mechanisms involve proteins that facilitate the aggregation of BK channels into functional clusters at the neuronal membrane

The study conducted by Sterken *et al* (89) presented an extensive transcriptional profile of *C. elegans* in response to EtOH exposure. Their study indicated that genes associated with detoxification processes, including cytochrome P450 enzymes and glutathione S-transferases, were notably upregulated, implying that EtOH exposure triggers a detoxification response in *C. elegans*. It was suggested that EtOH functions as a stressor, prompting a defensive reaction within the organism. This was evidenced by the increased expression of genes linked to stress response, such as HSPs and other molecular chaperones (90).

*Rodent studies.* Rodent studies provide comprehensive insight into the genetic, neurobiological and behavioral aspects of the interaction of alcohol with BK channels (91). Mice can be subjected to genetic engineering techniques to induce the knockout of specific genes, including those responsible for encoding BK channel subunits. This method enables researchers to investigate the influence of these particular genes on alcohol sensitivity, tolerance and consumption. Additionally, mice can be manipulated to express mutated or human variants of genes, thereby facilitating the exploration of the functional roles of various BK channel subunits in response to alcohol (91,92). The first mouse study included in the present review is from 2002; the most recent one was published in 2024, as presented below:

In 2002, Knott *et al* (93) explored the processes that underlie alcohol tolerance in neuro-hypophysial terminals, focusing on the role of ion channel plasticity. This plasticity relates to the capacity of ion channels to modify their function following prolonged alcohol exposure. Marked alterations in both calcium and potassium channels were revealed as a result of chronic alcohol exposure. Specifically, the functioning of voltage-gated calcium channels diminished while the activity of BK channels increased. The observed plasticity in ion channels stabilized neurotransmitter release from the neuro-hypophysial terminals during chronic alcohol exposure, thereby ensuring the maintenance of essential physiological processes regulated by these terminals, such as vasopressin and oxytocin (94).

Subsequently, 2 years later, Pietrzykowski *et al* (95) used rat brain slices as the experimental model to study BK channels in CNS terminals. Their study identified two main components contributing to alcohol tolerance in BK channels: i) Decreased EtOH potentiation; and ii) decreased channel density (95). Chronic EtOH exposure reduced the ability of EtOH to enhance BK channel activity. This suggests a desensitization or adaptation of the channel to the potentiating effects of EtOH over time. In addition, chronic EtOH exposure reduced the number of functional BK channels in CNS terminals. These findings suggest that alcohol tolerance in CNS BK channels involves adaptive changes in channel function (EtOH potentiation) and structural changes (channel density). Understanding these mechanisms provides insight into the way the CNS adapts to chronic alcohol exposure and may inform therapeutic strategies for alcohol-related disorders (96).

Velázquez-Marrero *et al* (97) found in 2011 that the persistence of molecular tolerance to alcohol was markedly non0linear concerning the duration of the initial alcohol exposure. Short periods of exposure resulted in transient tolerance, while longer periods of exposure led to more persistent tolerance. Molecular adaptations in BK channels, including alterations in their phosphorylation state and interactions with other proteins, were observed following prolonged alcohol exposure. These changes contribute to the persistent nature of tolerance. Upon brief exposure to alcohol, a temporary tolerance is developed, which rapidly diminishes after alcohol consumption ceases. By contrast, prolonged exposure leads to enduring modifications in channel function, resulting in persistent tolerance even after alcohol is no longer present (97).

Magnesium (Mg²^+^) protects against the EtOH potentiation of BK channel activity. The presence of Mg²^+^ diminishes the potentiating effect of EtOH on BK channel activity, indicating a modulatory function for Mg²^+^ in the impact of EtOH on BK channels. Electrophysiological recordings have shown that in the absence of Mg²^+^, EtOH notably increases the likelihood of BK channel openings. However, this effect is mitigated in the presence of Mg²^+^, highlighting the interplay between Mg²^+^ and EtOH in regulating BK channel activity (98).

In 2015, Kreifeldt *et al* (99) concluded that the BK channel β1 subunit plays a marked role in the behavioral adaptations elicited by chronic intermittent EtOH exposure. The presence of the β1 subunit contributes to regulating anxiety-like behavior, EtOH consumption and locomotor activity in response to EtOH. Furthermore, Palacio *et al* (100) concluded that EtOH exerts time-dependent effects on the expression and trafficking of BK channels in hippocampal neurons. Acute exposure increases BK channel surface expression and activity, while chronic exposure results in a more complex regulation that decreases surface expression after prolonged exposure. These findings highlight the dynamic nature of the impact of EtOH on ion channel regulation and provide insight into the molecular mechanisms underlying the neuronal adaptations to alcohol exposure.

The 2024 study focused on the residue in the α subunit of the BK channel, specifically hypothesizing the significance of this residue in the EtOH modulation of the channel and the subsequent behavioral responses to alcohol. Upon comparing the effects of EtOH on both wild-type and mutant mice regarding the specified residue, Okhuarobo *et al* (101) found no substantial differences in their behavioral responses. This suggests that other residues or mechanisms are likely accountable for the effects of EtOH on behavior. Consequently, this calls for further exploration of potential sites or mechanisms within the BK channel or other molecular pathways implicated in alcohol responses (101).

*Cell line studies.* Cell lines provide a controlled and consistent environment for studying the effects of EtOH on BK channels. They can be genetically manipulated easily to express various BK channel subunit isoforms. Using a cell line allows for the application of electrophysiological techniques, such as patch-clamp recordings, to measure the activity of BK channels under different conditions. This provides detailed information about the way EtOH affects channel function depending on subunit composition (102).

In 2008, Feinberg-Zadek *et al* (103) investigated the influence of BK channel subunit composition on the acute effects of alcohol. The findings revealed that the β4 subunit reduces alcohol-induced potentiation, while the β1 subunit has an even stronger effect in diminishing the impact of alcohol on BK channel activity. Additionally, the β subunits markedly affected the channel's plasticity in response to EtOH. Specifically, channels containing the β1 subunit exhibited higher initial sensitivity to EtOH but developed tolerance more rapidly than those containing other β subunits (103). The same year, Martin *et al* (104) also presented an auxiliary protein, the β4 subunit, associated with the BK channel. Electrophysiological recordings revealed that BK channels containing the β4 subunit exhibited reduced EtOH-induced activation compared with channels without the β4 subunit. It opened potential avenues for therapeutic strategies targeting the BK channel-β4 interaction to address alcohol-related disorders. In addition, the gene that encodes the BK channel β4 subunit should be considered a potential candidate for evaluating susceptibility to alcohol abuse and alcoholism (105). This suggests critical functional consequences for alcohol tolerance, as subunit composition may influence how neurons and cells adapt to alcohol exposure.

According to Yuan *et al* (106), specific lipids such as cholesterol and phosphatidylinositol 4,5-bisphosphate, influence the response of the channel to EtOH. For example, cholesterol-enriched membranes exhibit a different acute tolerance pattern compared with cholesterol-depleted membranes. The authors found that lipid composition notably modulates the development of alcohol tolerance in BKCa channels (106). Specifically, the lipid environment around the channel alters how the channel responds to alcohol. When the lipid composition was changed, it influenced the degree of potentiation and tolerance to alcohol, indicating that lipids are essential in modulating the channel's functional response to alcohol. That study highlighted that lipids can either enhance or reduce the development of alcohol tolerance in BKCa channels. This suggests that lipids are not just passive structural components, but actively regulate the way the BKCa channel adapts to EtOH exposure (105,106).

Pietrzykowski *et al* (107) elucidated a novel mechanism through which chronic alcohol exposure leads to neuroadaptive changes in neurons. Their study provided insight into the molecular processes involved in alcohol-induced neuroadaptation by identifying microRNA (miR)-9 as a key regulator of BK channel splice variant stability. Increased levels of miR-9 were observed in neurons following chronic alcohol exposure. This post-transcriptional regulation allows neurons to adjust their excitability and function in response to prolonged alcohol exposure.

Using patch-clamp recordings, Sitdikova *et al* (108) demonstrated that hydrogen sulfide (H_2_S) directly increased the open probability of BK channels. The effect of H_2_S on BK channel activity was mediated through direct interaction with the channel protein rather than through changes in intracellular signaling pathways. This conclusion was drawn from experiments demonstrating that the effect of H_2_S persisted even in the absence of intracellular calcium changes. That study demonstrated that H_2_S notably enhanced the activity of BK channels in rat pituitary tumor GH_3_ cells in a concentration-dependent manner (108). The effect is likely due to a direct interaction with the channel protein, increasing its open probability. That study paves the way for further research on the mechanisms through which H_2_S may influence ion channels in different cell types and contribute to processes, such as vascular tone regulation, smooth muscle function and neuronal excitability (108).

In 2013, Handlechner *et al* (109) demonstrated that EtOH and its metabolite acetaldehyde could increase the activity of BK channels in GH3 pituitary tumor cells. Combining these two substances produces a synergistic effect, leading to a more pronounced activation of BK channels than either substance alone. Subsequebntly, 1 year later, Velázquez-Marrero *et al* (110) showed that the β4 subunit affects the way PKA and PKC modulate the BK channel activity in the presence of alcohol. Channels with the β4 subunit showed a different pattern of tolerance development, suggesting that this subunit modulates how the channel adapts to prolonged alcohol exposure. The β4 subunit alters the mechanisms through which the channel responds to alcohol and kinase activity, affecting its voltage and calcium sensitivity. These findings suggest that individuals with different variants of the β4 subunit may experience varying degrees of tolerance to alcohol, which has important implications for understanding AUD and addiction. Targeting the interaction between the BK channel and kinases could provide novel therapeutic strategies for managing alcohol-related behaviors (110).

The interaction between Wnt/β-Catenin signaling and BK channels in the context of alcohol involves a multifaceted relationship. Alcohol can modulate both pathways, and this modulation can lead to changes in neuronal excitability and synaptic plasticity, and potentially contribute to the development of alcohol-related neuroadaptive changes. The β4 subunit has been found to influence phosphorylation states of the BK channel, which in turn affects the mechanisms through which the channel responds to alcohol and its subsequent tolerance (110,111).

In the case of BK channels, kinase-induced phosphorylation alters the gating and conductance, which can affect the mechanisms though which the channel responds to stimuli such as alcohol. Kinases modify BK channel activity over time, including their response to alcohol. When alcohol is repeatedly consumed, kinase-mediated phosphorylation can alter the function of the channels, making them less responsive to alcohol. The interaction between kinases and BK channels could contribute to individual differences in alcohol sensitivity and the development of alcohol-related disorders such as AUD. Targeting the kinases involved in BK channel modulation may provide novel approaches for the treatment of management of alcohol addiction by either promoting or inhibiting specific kinase pathways (109,110).

In 2016, Velázquez-Marrero *et al* (111) demonstrated that alcohol-induced increases in BK channel surface expression are dependent on the Wnt/β-catenin signaling pathway. Inhibiting this pathway blocked the alcohol-induced enhancement of BK channels, which in turn affected neuronal excitability by altering membrane potential and firing properties. Alcohol exposure also led to the internalization of BK channels, a process mediated by Wnt/β-catenin signaling and requiring new protein synthesis. Increased β-catenin levels from alcohol exposure inhibited this internalization, highlighting the role of the Wnt/β-catenin pathway in alcohol-induced neuronal adaptations and potential development of alcohol tolerance (111).

The 2024 study by Padilla *et al* (112) demonstrated that miR-9 binds to specific sites within the 3'UTRs of the BK ZERO isoform mRNA. This binding negatively regulates the expression of the BK channels by decreasing mRNA stability and inhibiting translation, potentially impacting understanding mechanisms underlying neuronal function and plasticity (113).

In another study, immunocytochemistry revealed that cells expressing the 2.1 3'UTR construct had markedly reduced BK channel expression when treated with miR-9, indicating responsiveness to miR-9. By contrast, cells with the 2.2 3'UTR or control constructs did not exhibit notable changes in BK channel expression upon miR-9 treatment, confirming their unresponsiveness (112).

### Contradictory findings observed between studies

Some pro- and anti-tolerance evidence has been reported. Studies on *Drosophila* and mammals have suggested that BK channels contribute to the development of tolerance by altering neuronal excitability after alcohol exposure (59,64,66,76). Other studies have reported that alcohol inhibits BK channel activity, suggesting a role in acute alcohol sensitivity rather than in developing tolerance (68,73,77). Some studies have reported that EtOH directly enhances BK channel opening, increasing K^+^ efflux and leading to neuronal hyperpolarization (69,114). By contrast, other studies have demonstrated that EtOH reduces BK channel activity, leading to depolarization and increased excitability, which could impair the development of tolerance (56,62,71).

BK channels are heteromeric, composed of pore-forming α subunits and modulatory β or γ subunits, influencing channel sensitivity to EtOH. Channels with certain β subunits (β4) show increased EtOH sensitivity, supporting a role in tolerance. Other subunit compositions (lack of β subunits) show diminished EtOH effects or even opposite effects. Variability in subunit expression across tissues and species complicates the interpretation of the impact of EtOH on BK channels (73-79).

In *Drosophila*, BK channel mutations can enhance or suppress alcohol tolerance, depending on the mutation type and experimental conditions. In rodents, BK channels have been implicated in both promoting and impairing alcohol tolerance, depending on brain region and cell type. The diversity of findings across species and brain regions challenges generalizing results. Also, EtOH is reported to activate or inhibit BK channels transiently during acute exposure, with effects that depend on the concentration and duration of exposure (94,95).

### Measured behaviors in fruit flies vs. rodents in EtOH use. EtOH tolerance

Repeated exposure to EtOH increases BK channel expression or alters functionality in fruit flies and rodents. In fruit flies, EtOH tolerance is associated with the upregulation of Slo gene transcription, which reduces the sedation effects over time. In rodents, specific β subunits modify the speed of EtOH tolerance development, with β1 subunit expression in smooth muscle and β4 subunit expression in the brain playing key roles (113,114).

*Locomotion*. In fruit flies, the Slo gene is the primary determinant of BK channel activity, as flies have a simpler genetic structure without extensive subunit diversity. Mutations in the Slo gene reduce EtOH-induced sedation and impaired locomotion, suggesting a direct link between BK channel function and motor control. In rodents, the β4 subunit of BK channels is particularly implicated in cerebellar neurons, which regulate motor coordination. Loss of β4 leads to exaggerated locomotor impairment following EtOH exposure, indicating a role in EtOH-induced motor deficits (115).

Withdrawal symptoms, such as tremors or hyperactivity, are observed primarily in rodents. The γ1 subunit of BK channels, found in brain regions such as the hippocampus and amygdala, modulates withdrawal symptoms. An enhanced expression of γ1 subunit is associated with reduced withdrawal severity, suggesting it mitigates hyperexcitability during EtOH abstinence. In fruit flies, withdrawal behaviors are less pronounced, but may manifest as increased locomotor activity post-EtOH exposure. Changes in Slo gene expression modulate these effects (116).

*Behavioral sensitivity*. The γ2 subunit in rodents enhances EtOH sensitivity by decreasing the threshold for BK channel activation. Mice lacking the γ2 subunit exhibit reduced sedation and tolerance. In fruit flies, behavioral sensitivity is directly linked to the phosphorylation state of the Slo-encoded α subunit, which modifies channel gating in response to EtOH. Comparing specific subunit compositions of BK channels across species reveals different regulatory roles. In fruit flies, behavioral changes are predominantly mediated by the α subunit encoded by Slo. In rodents, the presence and distribution of auxiliary β and γ subunits diversify EtOH responses, influencing behaviors like locomotion, tolerance and withdrawal symptoms (117).

The interaction of EtOH with BK channels indicates conserved molecular mechanisms across fruit flies and rodents, particularly in channel modulation and behavioral impacts. However, differences in neural complexity, channel subunit diversity and metabolic factors lead to distinct EtOH sensitivity and behavioral outcomes. These differences highlight the value of each model in studying specific aspects of alcohol tolerance and dependence.

Research on alcohol tolerance and BK channels is a developing field that merges neurobiology, pharmacology and genetics. Studies across various cell lines and animal models, including *C. elegans*, fruit flies and rodents, have indicated that alcohol modulates BK channel activity, influencing both acute and chronic behavioral responses to alcohol (117). Genetic variants in BK channel genes have been linked to differences in alcohol sensitivity, highlighting individual variability. Parker *et al* (118) suggested that similarities in alcohol sensitivity patterns between rodents and humans support the use of animal models as translational endophenotypes. Additionally, studies (119-121), including the one by Rinker and Mulholland (120) identified genes and pathways that affect AUD risk and treatment responses, utilizing genetically modified rodent models to explore the biological mechanisms behind the disorder.

The study by Yang *et al* (122) examined hispidol, a natural compound that influences behavioral responses to EtOH by acting on large-conductance calcium- and voltage-activated potassium BK channels. Hispidol targets the α subunit of the BK channel, potentially normalizing its activity affected by alcohol. In animal models, it reduced alcohol intoxication symptoms such as motor impairment and sedation, and alleviated anxiety-like behaviors during withdrawal. These findings suggest that hispidol may be a promising treatment for AUD, addressing both acute and withdrawal symptoms through its dual action on BK channels (122).

Addressing the challenges in translating preclinical findings to human studies is essential, highlighting the urgent need for further research to validate these targets in clinical trials. By modulating BK channel activity, it may be possible to reduce alcohol tolerance and withdrawal symptoms, helping individuals to reduce or stop alcohol consumption. In addition, this therapeutic angle is grounded in the focus of psychobiology on applying biological insights to improve mental health outcomes, highlighting the relevance of BK channels in developing new treatments for AUD.

The study by Pietrzykowski *et al* (107) provided key insight into the molecular mechanisms by which alcohol influences BK channel expression and function; however, it did not directly assess the behavioral outcomes associated with these molecular changes. Therefore, the connection between the observed molecular adaptations and specific behavioral manifestations of alcohol exposure or tolerance was not explicitly established within that study. Salkoff *et al* (123) discussed the structural modulation of BK channels, although with less emphasis on the effects of the lipid bilayer. The studies by Dopico *et al* (20,102,114) demonstrated the conserved roles of BK channels in EtOH responses across species and highlighted the way the surrounding lipid environment influences the effect of EtOH on BK channels. They uniquely highlighted the role of the lipid environment (cholesterol and phospholipids) in altering the impact of EtOH on BK channels. The studies by Dopico *et al* (20,102,114) highlight the conservation of EtOH-BK channel interactions across species and tissues, while other reviews have primarily focused on neuronal or vascular smooth muscle systems (107,123).

### Limitations of the current literature

Current literature on BK channels and alcohol tolerance has made notable progress; however, several limitations need to be addressed. While studies have identified that BK channels play a role in alcohol sensitivity and tolerance, the precise molecular mechanisms by which EtOH interacts with BK channels remain unclear. For example, the exact binding sites or conformational changes induced by EtOH on BK channels are not yet fully understood. The role of different auxiliary subunits (such as γ2, β1 and β4) in modulating EtOH effects is still being unraveled, and their interactions are not yet well-characterized (20,23,24).

The majority of research has been conducted in rodent models, which may not fully translate to humans. Rodent BK channels may have structural or functional differences affecting their EtOH interaction. Although some behavioral outcomes such as sedation and tolerance are linked to BK channels, the connection between specific neural circuits, BK channel activity and complex behaviors influenced by alcohol such as addiction, withdrawal, or relapse is not yet well-defined (68,86,102).

BK channels do not operate in isolation, but interact with other ion channels, such as voltage-gated calcium channels, NMDA receptors and GABA receptors. The extent to which EtOH affects these interactions has not yet been extensively studied, leaving a gap in the understanding of the mechanisms through which BK channel modulation integrate with broader neuronal signaling. Although BK channels are implicated in EtOH sensitivity and tolerance, only a limited number of studies have translated this knowledge into clinical applications (102,114). The potential of BK channels as therapeutic targets for AUD treatment is promising, yet it remains underexplored. Identifying compounds that modulate BK channel activity without adverse effects remains a significant challenge.

In conclusion, BK channels modulate neuron firing rates by influencing the duration and frequency of action potentials. EtOH alters the function of BK channels, leading to changes in neuronal excitability and synaptic plasticity. These modifications contribute to the acute and chronic effects of alcohol on the brain, including the development of tolerance and dependence. EtOH interacts with BK channels by altering their gating properties and calcium sensitivity, resulting in adaptive neuron changes to maintain homeostasis when exposed to alcohol. These changes are associated with behavioral alterations observed in AUD, such as impaired motor coordination, sedation and the reinforcing effects of alcohol.

BK channels are a shared target of alcohol, rendering them a focal point for understanding tolerance across species. Studies on *Drosophila* and *C. elegans* provide insight into genetic and molecular mechanisms and mouse models bridge findings to mammalian physiology and behavior. Cell lines allow for mechanistic studies under controlled conditions. Targeting BK channels presents a promising therapeutic strategy for AUD. Modulating these channels could help mitigate the effects of alcohol on the brain, reduce tolerance development and potentially aid in the treatment of dependence. Ongoing research using model organisms and human genetic studies aims to uncover the detailed mechanisms by which BK channels contribute to AUD, which is essential for developing targeted interventions.

The present systematic review was limited to only two online databases and focused on articles regarding fruit flies, nematodes, rodents and cell lines. Due to differences in complexity and regulatory mechanisms, the findings in model organisms may not directly apply to human physiology. While cell lines offer controlled environments, they lack the complexity of whole organisms, limiting the applicability of the results to more complex biological systems. Even though a meta-analysis was not conducted and additional research is required, the present systematic review presents an overview of the implications of BK channels in alcohol tolerance on animal models and cell lines.

## Figures and Tables

**Table I:** Risk of bias.

A, The SYRCLE tool for assessing the risk of bias												
	Selection bias	Selection bias	Selection bias	Performance bias	Performance bias	Detection bias	Detection bias	Attrition bias	Reporting bias	Other		
First author, year of publication	D1 Sequence generation	D2 Baseline characteristics	D3 Allocation concealment	D4 Random housing	D5 Blinding	D6 Random outcome assessment	D7 Blinding	D8 Incomplete outcome data	D9 Selective outcome reporting	D10 Other sources of bias	Overall	(Refs.)
Ghezzi, 2004	Low	Low	Not applicable	Low	Unclear	Low	High	Unclear	Unclear	Unclear	Low	(57)
Cowmeadow, 2005	Low	Low	Not applicable	Low	Unclear	Low	High	Unclear	Unclear	Unclear	Low	(59)
Cowmeadow, 2006	Low	Low	Not applicable	Low	Unclear	Low	High	Unclear	High	Unclear	Low	(60)
Wang, 2007	Low	Low	Not applicable	Low	Unclear	Low	Unclear	Unclear	Unclear	Unclear	Low	(62)
Wang, 2009	Low	Low	Not applicable	Low	Unclear	Low	High	Unclear	Unclear	Unclear	Low	(64)
Ghezzi, 2010	Low	Low	Not applicable	Low	Unclear	Low	High	Unclear	Unclear	Unclear	Low	(66)
Krishnan, 2012	Low	Low	Not applicable	Low	Unclear	Low	High	Unclear	High	Unclear	Low	(68)
Ghezzi, 2013	Low	Low	Not applicable	Low	Unclear	Low	High	Unclear	Unclear	Unclear	Low	(69)
Li, 2013	Low	Low	Not applicable	Low	Unclear	Low	High	Unclear	Unclear	Unclear	Low	(71)
Ghezzi, 2014	Low	Low	Not applicable	Low	Unclear	Low	Unclear	Unclear	High	Unclear	Low	(73)
Li, 2015	Low	Low	Not applicable	Low	Unclear	Low	High	Unclear	Unclear	Unclear	Low	(74)
Krishnan, 2016	Low	Low	Not applicable	Low	Unclear	Low	High	Unclear	Unclear	Unclear	Low	(76)
Bettinger, 2012	Low	Low	Not applicable	Low	Unclear	Low	Unclear	Unclear	Unclear	Unclear	Low	(81)
Davis, 2014	Low	Low	Not applicable	Low	Unclear	Low	Unclear	Unclear	Unclear	Unclear	Low	(82)
Davis, 2015	Low	Low	Not applicable	Low	Unclear	Low	Unclear	Unclear	Unclear	Unclear	Low	(84)
Scott, 2017	Low	Low	Not applicable	Low	Unclear	Low	High	Unclear	Unclear	Unclear	Low	(48)
Chen, 2018	Low	Low	Not applicable	Low	Unclear	Low	High	Unclear	High	Unclear	Low	(86)
Oh, 2019	Low	Low	Not applicable	Low	Unclear	Low	High	Unclear	Unclear	Unclear	Low	(87)
Sterken, 2021	Low	Low	Not applicable	Low	Unclear	Low	Unclear	Unclear	Unclear	Unclear	Low	(89)
Knott, 2002	Low	Low	Not applicable	Low	Unclear	Low	Unclear	Unclear	Unclear	Unclear	Low	(93)
Pietrzykowski, 2004	Low	Low	Not applicable	Low	Unclear	Low	High	Unclear	Unclear	Unclear	Low	(95)
Velázquez-Marrero, 2011	Low	Low	Not applicable	Low	Unclear	Low	High	Unclear	Unclear	Unclear	Low	(97)
Marrero, 2015	Low	Low	Not applicable	Low	Unclear	Low	Unclear	Unclear	Unclear	Unclear	Low	(98)
Kreifeldt, 2015	Low	Low	Not applicable	Low	Unclear	Low	High	Unclear	Unclear	Unclear	Low	(99)
Palacio, 2015	Low	Low	Not applicable	Low	Unclear	Low	High	Unclear	Unclear	Unclear	Low	(100)
Okhuarobo, 2024	Low	Low	Not applicable	Low	Unclear	Low	Unclear	Unclear	Unclear	Unclear	Low	(101)
B, The risk of bias in non-randomized studies-of interventions (ROBINS-I)												
First author, year of publication	Bias due to confounding		Bias in selection of participants into the study	Bias in classification of interventions	Bias due to deviations from intended interventions		Bias due to missing data	Bias in measurement of outcomes	Bias in selection of the reported result		Overall	(Refs.)
Feinberg-Zadek, 2008	Moderate		Low	Low	Moderate		Low	Low	Moderate		Low	(103)
Martin, 2008	Moderate		Low	Low	Moderate		Low	Low	Moderate		Low	(104)
Yuan, 2008	Moderate		Low	Low	Moderate		Low	Low	Moderate		Low	(106)
Pietrzykowski, 2008	Moderate		Low	Low	Moderate		Low	Low	Moderate		Low	(107)
Sitdikova, 2010	Moderate		Low	Low	Moderate		Low	Low	Moderate		Low	(108)
Handlechner, 2013	Moderate		Low	Low	Moderate		Low	Low	Moderate		Low	(109)
Velázquez-Marrero, 2014	Moderate		Low	Low	Moderate		Low	Low	Moderate		Low	(110)
Velázquez-Marrero, 2016	Moderate		Low	Low	Moderate		Low	Low	Moderate		Low	(111)
Padilla, 2024	Moderate		Low	Low	Moderate		Low	Low	Moderate		Low	(112)

The risk of bias was assessed as follows: D1, Was the allocation sequence adequately generated and applied? D2, Were the groups similar at baseline or were they adjusted for confounders in the analysis? D3, Was the allocation adequately concealed? D4, Were the animals randomly housed during the experiment? D5, Were the caregivers and /or investigators blinded from knowledge which intervention each animal received during the experiment? D6, Were animals selected at random for outcome assessment? D7, Was the outcome assessor blinded? D8, Were incomplete outcome data adequately addressed? D9, Are reports of the study free of selective outcome reporting? D10, Was the study apparently free of other problems that could result in high risk of bias?

**Table II:** *Drosophila melanogaster* studies.

Study no.	First author, year of publication	Study aim	Methods and statistical analysis	Main results	(Refs.)
1.	Ghezzi, 2004	To investigate the role of the SLO K(+) channel gene in rapid tolerance to sedative drugs (Benzyl Alcohol).	- Benzyl Alcohol Behavioral Test (the log-rank test showed a significant difference from the control group with P<0.01). - The statistics determine if the recovery curves are significantly different. - Quantitative Real-Time PCR Analysis and β-Galactosidase (β-Gal) Assay: Student's t-test was used to calculate significance.	Benzyl alcohol intoxication enhances SLO mRNA levels, a response to sedation. Tolerance to benzyl alcohol relies on SLO expression, which also predicts drug sensitivity.	(57)
2.	Cowmeadow, 2005	To investigate how the slowpoke (SLO) gene, which encodes the BK channel, contributes to rapid ethanol tolerance.	- Tolerance=a significant leftward shift in the recovery curve from prior ethanol exposure, determined by log-rank survival analysis. - The statistics evaluated the entire population's recovery curves to determine significance. - Methods used to study how flies process ethanol: gas chromatography, an enzymatic test for ethanol and checking the flies' water content.	Rapid tolerance to ethanol in Drosophila, shown by a shorter sedation period, relies on the slowpoke gene's expression in the nervous system. Mutations in this gene eliminate rapid tolerance.	(59)
3.	Cowmeadow, 2006	To assess if ethanol sedation triggers slowpoke gene expression and if this expression alone can produce a phenotype resembling ethanol tolerance.	The log-rank test was used to compare the recovery curves of two groups of sedated flies in various behavioral tests, including ethanol and benzyl alcohol sedation, ethanol tolerance, cross- tolerance, heat shock, and ethanol resistance tests. - Quantitative Real-Time Polymerase Chain Reaction and β-Galactosidase and Protein Assays: Student's t-test was used to calculate significance.	Ethanol consumption elevates the levels of slowpoke mRNA, signifying a transcriptional response to the substance. Additionally, artificially inducing the expression of slowpoke confers resistance to the effects of ethanol.	(60)
4.	Wang, 2007	To investigate how repeated drug exposure causes stable gene expression and behavioral changes, focusing on histone modifications and DNA methylation.	- Tolerance assay: The significance of curves was assessed using the log-rank test. - Chromatin immunoprecipitation assay: A two-way ANOVA test was used to evaluate significance. - Quantitative RT-PCR analysis: Student's t-test was used to calculate significance.	Modifications in histone acetylation and DNA methylation, influenced by drug exposure, play a significant role in regulating gene expression related to neural adaptations for tolerance. Specifically, sedation with benzyl alcohol has been found to actively modify the acetylation state of the SLO control region. Additionally, benzyl alcohol sedation has been shown to promote the occupancy of dCREB2 in the SLO transcriptional control region, further influencing gene expression.	(62)
5.	Wang, 2009	To study the role of CREB in regulating SLO expression and rapid tolerance from benzyl alcohol sedation.	Tolerance assay: A log-rank test was used to determine whether there was a significant difference in recovery time between the two groups. - Chromatin immunoprecipitation: It was used a two-way ANOVA to determine significance. - Quantitative RT PCR: It calculated mRNA levels using the standard curve method and checked significance with Student's t-test.	Sedation induced by benzyl alcohol enhances the binding of phosphorylated dCREB2 to the promoter region of the SLO gene. A loss-of-function mutation in dCREB2 inhibits the induction of SLO, which is typically triggered by sedation. This mutation also removes the of the organism to develop rapid tolerance to anesthetic effects, underscoring the critical role of dCREB2 in these processes.	(64)
6.	Ghezzi, 2010	To investigate the role of BK channels in drug tolerance and dependence, specifically examining their influence as counter-adaptive mechanisms.	- Electrophysiological assay - Determination of neuronal following frequency: Two-way ANOVA and Student's t-test were used to calculate significance. - Determination of seizure susceptibility. - Behavioral and electrophysiological tolerance assay.	The induction of the SLO gene is a neuroadaptive process driving drug tolerance. In the absence of the drug, increased SLO expression creates an allostatic state that results in withdrawal symptoms, linking tolerance and withdrawal hyperexcitability in *Drosophila*. Benzyl alcohol-treated wild-type flies were more liable to seizures than untreated controls.	(66)
7.	Krishnan, 2012	To examine how dynamin contributes to the development of ethanol tolerance.	- Tolerance assay - A log-rank test was used to examine if the differences in time-to-event data are significant. - Heat-shock protocols - Ethanol plus heat shock - Quantification of mRNA - Student's t-test. - Measurement of ethanol concentrations using gas chromatography	The development of rapid functional tolerance to ethanol involves a critical interplay between the shibire gene and Syntaxin 1A. The shibire gene encodes Dynamin, a protein that plays a fundame- ntal role in the initial response to ethanol exposure. When ethanol is present, Dynamin activates a cascade of cellular events, ultimately leading to the swift adaptation of the cell, allowing it to tolerate higher concentrations of ethanol more effectively.	(68)
8.	Ghezzi, 2013	To identify and characterize the specific neuronal substrates and genetic mechanisms underlying drug tolerance.	- Quantification of UAS-slo transgene induction - Student's t-test. - Behavioral analysis of resistance to sedation-log- rank test.	Both the shibire gene and Syntaxin 1A are vital for developing rapid functional tolerance to ethanol. This suggests a significant interaction between these proteins and the body's adaptive mechanisms in response to alcohol.	(69)
9.	Li, 2013	To identify a DNA element that regulates drug tolerance and withdrawal genes.	- In the tolerance assay, the log-rank test assessed the significance of sedation recovery curves. One-way ANOVA with Dunnett's post hoc analysis was used for daily activity, walking speed, and climbing assays. - Chromatin immunoprecipitation (ChIP) assay: one-way ANOVA - For the analysis of reverse transcription- quantitative PCR and the changes in seizure threshold, it was used an unpaired Student's t-test to determine significance.	The Slo-delta6b mutants exhibit an exaggerated transcriptional response to sedative drugs, leading to an enhanced and prolonged physiological tolerance to such substances. This mutation in the Slo-delta6b gene contributes to an intensified sensitivity to drug sedation, resulting in a marked increase in the overall response of these mutants when exposed to sedative agents.	(71)
10.	Ghezzi, 2014	To explore the impact of BK channel gene expression on the vulnerability to seizures during ethanol withdrawal.	- Electrophysiological analysis of seizure susceptibility involved calculating the average seizure threshold for each group and assessing statistical significance with a Student's t-test. - Tissue dissection and acridine orange staining - Differences between ethanol and control groups were assessed using Student's t-test.	Research indicates that the expression levels of the BK channel gene play a crucial role in determining susceptibility to seizures during ethanol withdrawal. Specifically, higher expression of the BK channel gene has been linked to an increased frequency and severity of seizures associated with ethanol withdrawal.	(73)
11.	Li, 2015	To evaluate the 55b element's role in baseline SLO expression, its response to benzyl alcohol sedation, and the effects of its removal.	- Ends-out gene targeting of 55b element - Southern blotting - Benzyl alcohol tolerance/resistance assays - Quantitative RT-PCR - Chromatin immunoprecipitation - Climbing assay (Student's t-test for significance) - Flight and sticky-feet assays - Circadian rhythm and activity measurement - Electrophysiological analysis (Student's t-test for significance)	The study identified a specific DNA element within the SLO gene associated with histone modifications indicative of active gene expression in muscle tissue. - The 55b element plays no essential role in the changes in H4 acetylation induced by benzyl alcohol. Additionally, the slo-delta55b variant does not influence behavioral responses related to benzyl alcohol, nor does it have any effect on circadian rhythmicity.	(74)
12.	Krishnan, 2016	To investigate how a particular DNA element within the SLO gene affects the development of ethanol tolerance.	- Functional tolerance assay: Curve separation was evaluated using log-rank analysis. Chromatin immunoprecipitation assay: Significance determined by one-way ANOVA with Dunnett's comparison.	Mapping the effects of ethanol on histone acety- lation represents an effective method for identifying DNA regulatory elements that facilitate the mediation of gene responses to ethanol exposure. It is proposed that the regulatory element known as 6b plays a significant role in the homeostatic modulation of SLO expression in response to sedation induced by organic solvents. This indicates that 6b may be essential in maintaining the balance of gene activity when influenced by such solvents, thereby contributing to the physiological adaptation processes of the organism.	(76)

SLO, potassium calcium-activated channel (KCNMA1; also known as Slo1); CREB, cAMP response element-binding protein; BK channels, large-conductance calcium and voltage-activated potassium channels.

**Table III:** *Caenorhabditis elegans* studies.

Study no.	First author, year of publication	Study aim	Methods and statistical analysis	Main results	(Refs.)
1.	Bettinger, 2012	To explore how the lipid environment in *C. elegans* membranes influences acute ethanol tolerance development.	- Worm husbandry - Genetic screen - Speed analysis: One-way ANOVA with Tukey's post hoc test - Acute functional tolerance (AFT) tested using an unpaired two-tailed t-test - Ethanol response: assessed by initial sensitivity, AFT development, and comparison with control strain.	The lipid composition of cellular membranes significantly affects the development of acute ethanol tolerance in *Caenorhabditis elegans*. Mutants with disruptions in lipid metabolism genes show varying rates and degrees of ethanol tolerance compared to wild-type organisms. The specific lipid composition influences neuronal responses to ethanol, impacting both initial sensitivity and the subsequent development of acute tolerance. This association highlights the role of lipid metabolism in alcohol-related behaviors in these organisms.	(81)
2.	Davis, 2014	To identify an amino acid residue in the BK channel that influences alcohol- induced behaviors in. nematodes	- Transgenesis and site-directed mutagenesis - Locomotion posture assay - Egg-laying response to ethanol and locomotion response to ethanol - Aldicarb analysis, cell transfection, confocal imaging and electrophysiology - Data analysis: Data passing the Shapiro-Wilk normality test were analyzed using t-tests or ANOVA and the Holm-Sidak method for post hoc comparisons. Data failing the test were analyzed using the Mann-Whitney rank sum test or Kruskal-Wallis ANOVA on ranks with Dunn's test for post hoc comparisons.	A specific conserved amino acid, phenylalanine at position 143 (F143), plays an essential role in the activation of the BK channel by ethanol within the slo-1 gene. When this phenylalanine residue is mutated to alanine (F143A), the channel loses its responsiveness to ethanol. However, this mutation does not impair the overall functionality of the channel, indicating that while phenylalanine is crucial for the of ethanol, it is not vital for the basic operations of the channel itself.	(82)
3.	Davis, 2015	To investigate how the calcium-binding domains of the BK potassium channel mediate ethanol-induced intoxication and activation.	- Transgenesis and site-directed mutagenesis - Neck curvature and overall body curvature assays - Egg-laying and locomotion responses to ethanol - Confocal microscopy and electrophysiology Data analysis: - If normal (Shapiro-Wilk test): t-tests or ANOVA; post hoc: Holm-Sidak method. - If not normal, used Mann-Whitney or Wilcoxon signed-rank tests, or Kruskal-Wallis ANOVA. - Ethanol-induced channel opening changes tested using a z-test.	The potential calcium-binding domains of the BK potassium channel in *Caenorhabditis elegans* are not critical for the activation of the channel induced by ethanol. Alterations or deletions within these domains do not impede the channel's ability to be activated by ethanol. Furthermore, the behavioral responses associated with ethanol intoxication in *C. elegans* remain largely unaffected when the calcium-binding domains of the BK channel are compromised.	(84)
4.	Scott, 2017	To investigate how SLO-1, a BK potassium channel, affects behavioral deficits in *Caenorhabditis elegans* after chronic ethanol withdrawal.	- Transgenesis - Ethanol treatment - Diacetyl-race assay and locomotion assay - Gas chromatography and confocal microscopy - Quantitative PCR Data analysis: Groups were compared using t-tests or ANOVA, with post hoc Holm-Sidak comparisons as needed.	Mutant strains that lack functional SLO-1 channels exhibit significantly heightened behavioral deficits following ethanol withdrawal compared to their wild-type counterparts. These withdrawal impairments become exacerbated as a result of diminished neuronal SLO-1 channel function. Furthermore, SLO-2, a distinct large-conductance potassium channel, contributes to the severity of withdrawal impairments through a mechanism that is dependent on SLO-1 channels.	(48)
5.	Chen, 2018	To explore how the GCY-35/GCY-36 guanylate cyclase pathway and the TAX-2/TAX-4 cyclic nucleotide-gated ion channel in oxygen sensory neurons contribute to the development of acute tolerance to ethanol.	- Assays of worm behavior in response to acute ethanol treatment. - Chemogenetics. - Data analysis: One-way ANOVA tested mean values among three or more samples. Two-way ANOVA assessed differences between groups for time and genotypes. Bonferroni's correction was applied for multiple comparisons.	Research has shown that the signaling pathways of the GCY-35 and GCY-36 guanylate cyclases, along with the TAX-2 and TAX-4 ion channels, play a crucial role in the O_2_ sensory neurons of *C. elegans* for the development of acute functional ethanol tolerance. When these signaling pathways are disrupted, the nematodes struggle to adapt to exposure to ethanol, indicating their importance in the response of the organism to this substance.	(86)
6.	Oh, 2019	To explore how the clustering of BK channels influences behavioral responses to alcohol.	- Ethanol-resistant locomotory and egg-laying behavior assays - Microscopy and time-lapse imaging - Western blotting - Data analysis: Speed and egg-laying data were analyzed using one-way ANOVA with Tukey's post hoc; fluorescence clusters were analyzed with a two-tailed t-test.	Mutations in genes associated with the clustering of BK channels have been shown to affect the behavior of organisms when exposed to alcohol. The disruption of BK channel clustering results in modified sensitivity to alcohol. This study has identified the STOML-1 protein as a critical component in the clustering of BK channels.	(87)
7.	Sterken, 2021	To investigate the transcriptional response of *Caenorhabditis elegans* to ethanol exposure and the molecular mechanisms behind its reaction to alcohol.	- Nematode husbandry and age synchronization - RNA isolation, cDNA synthesis, labeling, microarray hybridization, and data extraction - Normalization and data transformation - ANOVA determined statistical significance; a linear model assessed gene expression affected by variables. - K-means clustering identified time-dependent patterns in differentially expressed genes.	Pathway analysis provided insight into the intricate biological processes affected by the differentially expressed genes, revealing that several critical pathways were significantly enriched. Among these were pathways involved in metabolic regulation, cellular stress response, and various signaling mechanisms essential for cell communication and function. The analysis showed a substantial upregulation of genes associated with the stress response following exposure to ethanol, highlighting the cellular adaptations triggered by this condition. This upregulation underscores the effort of the organism to manage oxidative stress and potential cellular damage.	(89)

SLO, potassium calcium-activated channel (KCNMA1; also known as Slo1); BK channels, large-conductance calcium and voltage-activated potassium channels.

**Table IV:** Rodent studies.

Study no.	First author, year of publication	Study aim	Rodent type	Methods and statistical analysis	Main results	(Refs.)
1.	Knott, 2002	To investigate the mechanisms under- lying alcohol tolerance in neurohypophysial terminals, focusing on the role of ion channel plasticity.	Male Sprague- Dawley rats	- The neurohypophysis was removed and placed in low-calcium Locke's solution. Hormones were collected from isolated neurohypophysial terminals, and potassium currents were measured using the whole-cell patch-clamp technique. Data analysis used ANOVA to evaluate differences between naive and long-term ethanol exposure groups.	Neurohypophysial terminals from alcohol- tolerant rats exhibited a reduced response to acute alcohol effects compared to non- tolerant rats. Inhibiting protein inase C (PKC) activity prevented alcohol-induced changes in BK channels, highlighting the critical role of PKC in this adaptive process.	(93)
2.	Pietrzykowski, 2004	To investigate alcohol tolerance mechanisms in large-conductance, calcium-activated potassium (BK) channels in CNS terminals.	Male Sprague- Dawley adult rats	- Viability assay - Neurohypophysial terminal preparation - Electrophysiology: Whole-cell and single- channel recordings - Superfusion and drug application - Immunocytochemistry: Confocal analysis and BK expression quantification - Data analysis: Student's t-test for statistical significance between treatment groups.	The phenomenon of alcohol tolerance in large conductance calcium-activated potassium (BK) channels is primarily characterized by two components: diminished ethanol potentiation and a reduction in channel density. Chronic exposure to alcohol has been associated with a notable decrease in the potentiation, or enhancement, of BK channel activity by ethanol. Furthermore, this prolonged exposure results in a decreased density of BK channels located in the plasma membrane of neurohypophysial terminals.	(95)
3.	Velázquez- Marrero, 2011	To investigate the nonlinear association between the length of initial alcohol exposure and the long-term changes in BK channel function associated with alcohol tolerance.	Primary cultures from mouse hippocampi	- Primary striatal culture methods: - 293 cell transfection - Electrophysiological recordings - In vivo ethanol exposure in P25-P33 male C57BL/6J mice - Acute dissociation of striatal neurons - Neuron survival assay - Immunocytochemistry - Reverse transcription PCR (significance via one-way ANOVA).	Prolonged initial exposures to alcohol result in more enduring tolerance, whereas brief exposures lead to less persistent or reversible alterations. Neurons subjected to extended durations of alcohol exposure demonstrate more significant and long- lasting effects on BK channel activity compared to those subjected to shorter exposure periods.	(97)
4.	Marrero, 2015	To investigate the modulatory role of magnesium in the interaction between ethanol and BK channels.	Hippocampal CA1 neurons from embryonic mice	- Patching Procedure - Data Gathering - Tests with specific NPo values (NPo measures channel activity). - NPo as a function of voltage. Two-way ANOVAs performed for these tests. - Significant differences considered at P<0.05.	Elevated concentrations of magnesium have been shown to diminish the enhancing effect of ethanol on BK channel activity. This modulatory effect of magnesium is characterized by a dose-dependent association. It is plausible that magnesium influences the binding affinity or conformational state of BK channels in the presence of ethanol.	(98)
5.	Kreifeldt, 2015	To examine the role of the BK channel β1 subunit in behavioral adaptations to chronic intermittent ethanol (CIE) exposure.	BK β1 and β4 knockout (KO) mice (C57BL/6J background)	- Ethanol metabolism: - Effects: Ataxia, acute functional tolerance, hypothermia, sedation, handling-induced convulsions, and chronic intermittent exposure. Data analysis: One-way or repeated- measures ANOVA assessed the effects of genotype and ethanol treatment on intoxication durations. Handling-induced convulsions and AUC were analyzed using Kruskal-Wallis tests.	Mice that possess a genetic deletion of the BK channel β1 subunit, termed β1 knockout mice, exhibit notable alterations in their behavioral responses to chronic intermittent exposure to ethanol when compared to wild-type mice. β1 knockout mice demonstrate a reduced behavioral tolerance to ethanol, suggesting diminished adaptability to its effects. This research underscores the importance of the BK channel β1 subunit in influencing the behavioral effects of ethanol.	(99)
6.	Palacio, 2015	To study the effects of ethanol on BK channel expression and trafficking in hippocampal neurons over time.	Primar hippo- campal culture from E18 mouse hippocampal tissue	- Immunofluorescence - TIRF imaging - Western blotting - Electrophysiology - Student's t-test analysis using GraphPad Prism software	Ethanol exposure has been observed to induce time-dependent alterations in the expression levels of BK channels within hippocampal neurons. Its influence extends to the trafficking of these channels, affecting both their delivery to the cell surface and subsequent internalization processes. The impact of ethanol on the expression and trafficking of BK channels demonstrates a clear dependence on time.	(100)
7.	Okhuarobo, 2024	To investigate whether the interaction between ethanol and BK channel α subunit residue K361 influences alcohol- related behaviors in mice.	C57BL/6J mice	- Electrophysiological recordings - Tremor, motor coordination, and ethanol- induced ataxia - Ethanol-induced analgesia, sedation, and hypothermia - Ethanol-conditioned place preference - Chronic intermittent ethanol (CIE) vapor inhalation - Data analysis: Electrophysiological data were assessed with paired t-tests; tremor scores with Kruskal-Wallis ANOVA and Dunn's comparisons; genotype effects on body weight, ataxia, and sedation via one-way ANOVA.	The mutation reported in K361 did not lead to any significant changes in the voluntary consumption of ethanol by the mice, indicating that the mutation did not affect their drinking behavior. Moreover, when assessing locomotor activity after the administration of ethanol, both wild-type and mutant mice exhibited comparable levels of activity, suggesting that the mutation did not influence locomotion in the presence of ethanol. In addition, the presence of this mutation did not result in any noteworthy changes in the activity of BK channels within neuronal cells when exposed to ethanol, implying that the mutation does not alter the neurophysiolo- gical effects typically induced by ethanol.	(101)

**Table V:** Cell line studies.

Study no.	First author, year of publication	Study aim	Methods and statistical analysis	Main results	(Refs.)
1.	Feinberg-Zadek, 2008	To investigate how BK channel subunit compositions influence molecular tolerance to ethanol.	- Cell culture for 293 cell lines - Charybdotoxin treatment - Electrophysiological recording - Steady-state channel activity calculation - Experimental paradigms: Ethanol application, chronic exposure (24 h) for tolerance assessment - Data analysis: Student's paired t-test.	The BK channels are composed of various subunits, and the specific combination of these subunits influences the channel's behavior in the presence of ethanol. The β4 subunit has been identified as a crucial factor in modulating both ethanol sensitivity and tolerance. BK channels that incorporate the β4 subunit exhibit reduced sensitivity to ethanol and demonstrate a unique pattern of tolerance development when compared to channels that lack this subunit.	(103)
2.	Martin, 2008	To investigate the role of an auxiliary protein linked to the BK channel in developing tolerance to alcohol.	- Cell culture: - Slice preparation and isolated striatal neurons from mice - Electrophysiological recordings - Steady-State channel activity calculation - Behavioral experiments - Data analysis: One-way ANOVA, Tukey's post hoc tests.	The BK channel β4 subunit is instrumental in the regulation of alcohol tolerance, exerting influence at both the molecular and behavioral levels. The gene encoding the BK channel β4 subunit, referred to as KCNMB4, should be regarded as a significant candidate for assessing susceptibility to alcohol use disorders and alcoholism. Investigating the role of KCNMB4 may provide valuable insights into the biological mechanisms underlying alcohol tolerance and the risk of alcohol dependency.	(104)
3.	Yuan, 2008	To explore whether acute alcohol tolerance is a key characteristic of BK channels and how the lipid environ- ment affects it.	- Electrophysiology: 293 cell recordings, planar bilayer recording - Ethanol analysis - Data analysis: nPo=steady-state channel activity from histogram, number of channels (n), and open channel probability (Po).	The large conductance calcium-activated potassium (BKCa) channel exhibits an inherent capacity to develop acute tolerance to alcohol. This adaptation occurs due to repeated exposure, leading to a diminished response of the BKCa channels to alcohol's effects. The lipid environment surrounding these channels is a critical factor in this process, as it significantly influences the modulation of acute alcohol tolerance. The specific composition and properties of these lipids play an essential role in determining the functional behavior of the BKCa channels in response to alcohol exposure.	(106)
4.	Pietrzykowski, 2008	To explore how microRNA-9 (miR-9) regulates the stability of BK channel splice variants and its role in neuroadaptation to alcohol.	- 293 cell transfection - Electrophysiology - Computational modeling of miR-9 regulation of BK transcripts - Data analysis included assessing distribution via frequency histograms, performing a Kolmogorov-Smirnov test for normality, and using ANOVA to compare treatment means.	The research highlighted the importance of miR-9, a specific microRNA, in regulating the stability of certain splice variants of the BK (big potassium) channel. The study revealed that chronic exposure to alcohol led to noticeable alterations in the levels of these particular BK channel variants. This finding indicates that the regulatory role of miR-9 may be a crucial mechanism enabling neurons to adapt to the sustained presence of alcohol over time.	(107)
5.	Sitdikova, 2010	To study how hydrogen sulfide (H_2_S) affects calcium-activated potassium (BK) channels in rat pituitary tumor (GH3) cells.	- GH3 pituitary cells - Electrophysiology - Chemicals and solutions - Data analysis: Student's t-test or one-way ANOVA with the Bonferroni correction as needed.	Hydrogen sulfide (H_2_S) significantly enhances the activity of large-conductance calcium-activated potassium (BK) channels. This enhancement exhibits a dose-dependent relationship, whereby increasing concentrations of H_2_S lead to a more substantial increase in channel activity. Consequently, higher levels of H_2_S amplify the functional performance of BK channels and may have broader implications for various physiological and cellular processes that depend on these channels.	(108)
6.	Handlechner, 2013	To study how acetalde- hyde and ethanol affect BK channels in GH3 pituitary tumor cells.	- GH3 pituitary tumor cells - Solutions - Electrophysiology - Statistical analysis: Student's t-test, ANOVA with Bonferroni correction; significance at P<0.05.	Both ethanol and acetaldehyde have distinct effects on the activity of large-conductance calcium-activated potassium (BK) channels in GH3 pituitary tumor cells. Specifically, ethanol has been found to enhance the activity of these channels, leading to increased potassium ion conductance. By contrast, when applied in isolation, acetaldehyde reduces the activity of BK channels, resulting in decreased potassium ion flow. When acetaldehyde is present alongside ethanol, it counteracts the stimulating effect of ethanol on the BK channels, effectively negating the activation that ethanol alone would induce.	(109)
7.	Velázquez- Marrero, 2014	To investigate how the β4 subunit of BK channels affects alcohol sensitivity and tolerance through kinase modulation.	- Cell culture and transfection - Immunoprecipitation, SDS-PAGE, immunoblotting - Dissociated nucleus accumbens neurons - Bathing aolutions - Electrophysiological eecordings - Steady-state channel activity calculation - Data Analysis: Paired t-tests, P<0.05 significant.	Channels containing β4 showed altered responses to ethanol compared to channels without β4, indicating a regulatory role of this subunit in ethanol-induced effects. Modulation of PKA and PKC activity affected the ethanol sensitivity of β4-containing BK channels, suggesting that kinase-mediated phosphorylation is essential in regulating channel function in response to ethanol.	(110)
8.	Velazquez- Marrero, 2016	To explore how Wnt/β-catenin signaling influences the surface expression of BK channels when exposed to alcohol.	- Primary neuronal culture (embryonic Sprague-Dawley rat hippocampal tissue) - Immunocytochemistry - Isolation of synthesized proteins - Cellular fractionation and immunoblotting - Electrophysiology - Proteomics - Data analysis: Student's t-test or one-way ANOVA with Dunnett's post hot test (P<0.05 for significance).	The increase in the surface expression of BK channels induced by alcohol has been demonstrated to be mediated through the Wnt/β-catenin signaling pathway. The activation of this pathway by alcohol involves the stabilization and subsequent nuclear translocation of β-catenin.	(111)
9.	Padilla, 2024	To investigate how microRNA-9 (miR-9) regulates BK ZERO isoform expression in 293 cells.	- 293 cells - Transfection - Monoclonal selection - Electrophysiology - Immunocytochemistry - Data analysis: BK expression in response to miR-9 was assessed via one-way ANOVA and Tukey's post hoc test.	MicroRNA-9 (miR-9) exhibits a differential regulatory effect on the expression of the BK ZERO isoform across 293 cells, dependent on the presence of specific 3' untranslated regions (3'UTRs) in the corresponding transcripts. By establishing stable cell lines featuring distinct 3'UTRs, researchers demonstrated that the binding of miR-9 to these regions diminishes the mRNA stability of BK channels and subsequently reduces protein expression. This interaction ultimately leads to significant alterations in potassium currents.	(112)

BK channels, large-conductance calcium and voltage-activated potassium channels.

## Data Availability

The data generated in the present study may be requested from the corresponding author.
